# Vitamin D binding protein (VDBP) hijacks twist1 to inhibit vasculogenic mimicry in hepatocellular carcinoma

**DOI:** 10.7150/thno.90322

**Published:** 2024-01-01

**Authors:** Lu-ning Qin, Heng Zhang, Qing-qing Li, Ting Wu, Shan-bin Cheng, Kai-wen Wang, Yue Shi, Hao-ran Ren, Xue-wu Xing, Cheng Yang, Tao Sun

**Affiliations:** 1 State Key Laboratory of Medicinal Chemical Biology and College of Pharmacy, Nankai University, Tianjin, China.; 2 Tianjin International Joint Academy of Biomedicine, Tianjin, China.; 3 Department of Orthopedics, Tianjin First Central Hospital, Tianjin, China.

**Keywords:** VDBP, HCC, VM, VDR, YY1

## Abstract

**Rationale:** Vitamin D (VD) has been suggested to have antitumor effects, however, research on the role of its transporter vitamin D-binding protein (VDBP, gene name as *GC*) in tumors is limited. In this study, we demonstrated the mechanism underlying the inhibition of vasculogenic mimicry (VM) by VDBP in hepatocellular carcinoma (HCC) and proposed an anti-tumor strategy of combining anti-PD-1 therapy with VD.

**Methods:** Three-dimensional cell culture models and mice with hepatocyte-specific *GC* deletion were utilized to study the correlation between VDBP expression and VM. A patient-derived tumor xenograft (PDX) model was further applied to validate the therapeutic efficacy of VD in combination with an anti-PD-1 drug.

**Results:** The study revealed that VDBP expression is negatively correlated with VM in HCC patients and elevated VDBP expression is associated with a favorable prognosis. The mechanism studies suggested VDBP hindered the binding of Twist1 on the promoter of VE-cadherin by interacting with its helix-loop-helix DNA binding domain, ultimately leading to the inhibition of VM. Furthermore, VD facilitated the translocation of the vitamin D receptor (VDR) into the nucleus where VDR interacts with Yin Yang 1 (YY1), leading to the transcriptional activation of VDBP. We further demonstrated that the combination of VD and anti-PD-1 led to an improvement in the anti-tumor efficacy of an anti-PD-1 drug.

**Conclusion:** Collectively, we identified VDBP as an important prognostic biomarker in HCC patients and uncovered it as a therapeutic target for enhancing the efficacy of immune therapy.

## Introduction

The vitamin D binding protein (VDBP) is well known for its role in transporting the VD metabolites 25OHD and 1,25OHD *in vivo* and maintaining their balance during bone metabolism. Over the years, VDBP has been revealed to also be closely associated with malignancies, including breast, prostate, pancreatic, lung, colorectal, and basal cell cancers, as well as cutaneous melanoma 1, 2. VDBP, a member of the albumin superfamily of binding proteins (albumin, methemoglobin, alpha-albumin/alfamin), is mainly expressed in the liver 3, 4, and can potentially affect the level of vitamin D metabolites, which may result in the development of disease 5. However, there is limited literature concerning the role of VDBP in hepatocellular carcinoma (HCC), and their correlation is not yet clear. In this work, we found, by immunohistochemical (IHC) analysis of 96 clinical HCC patients combined with TCGA database analysis, that VDBP is lowly expressed in HCC but highly expressed in adjacent normal tissues. Moreover, the expression of VDBP is negatively correlated with a more advanced clinical grade and stage of HCC. The main function of VDBP is related to macrophage activation and neutrophil chemotaxis, which are involved in immune regulation. In addition, its circulating concentration may also be associated with inflammatory regulation 6-8. VDBP has previously been reported to also have functions unrelated to VD transport, including binding of fatty acids and actin monomers, which is released in large amounts outside the vasculature by cell death and inhibits the occlude of vasculature caused by the F-actin network. Here, we report a novel function of VDBP, which interacts with Twist1, a key epithelial-mesenchymal transition (EMT) transcription factor, to inhibit vasculogenic mimicry (VM), a form of angiogenesis found in many malignancies that is not dependent on vascular endothelial cells in HCC 9. VM is commonly found in highly aggressive, highly metastatic, and advanced malignancies and usually suggests poor prognosis for patients 10. Vessels formed during VM consist of an arrangement of tumor cells with endothelial cell characteristics, these tubes deliver nutrients and oxygen-carrying red blood cells to the tumor 11, in which the presence of CD31/CD34-negative and PAS-positive cells and red blood cells are usually used as identification criteria for VM 12, 13. The expression of the transcription factor Twist1 is upregulated in tumor cells during VM, leading to the downregulation of the tight junction protein E-cadherin between epithelial cells and the significant upregulation of the VM-associated molecule VE-cadherin, which plays an extraordinarily crucial role in VM 14. Meanwhile, cancer cells produce large amounts of matrix metalloproteinases (MMPs) for the degradation of extracellular matrix components and basement membrane, providing space for VM and reducing resistance to cellular morphological changes during VM thereby promoting the invasiveness of tumor cells 15, 16. Twist1 has been reported to directly bind to the promoter region of VE-cadherin, a key marker of VM, to activate VE-cadherin expression to promote VM 17. It was found in the study that VDBP binding to Twist1 inhibits the binding of Twist1 to the VE-cadherin promoter, thus suppressing VM.

There is increasing clinical evidence proving that VD plays an important role in anti-tumor mechanisms 18; VD supplementation has been associated with reduced cancer mortality 19. The biological function of VD is mainly achieved by binding to the vitamin D receptor VDR. The VD-VDR complex binds to the vitamin D response element (VDRE) on the promoter of target genes to regulate gene expression 20. The VD-VDR signaling pathway is associated with the development of liver disease, and is an important factor driving the inflammatory process and liver injury 21, its deficiency is associated with the severity of liver disease 22. In this study, we revealed a novel anti-tumor mechanism of the VD-VDR axis through the regulation of VDBP levels, providing new insights into the field of oncology research.

Cancer immunotherapy has drawn increasing attention in the field of oncology, and antibodies targeting the PD-1/PD-L1 pathway have been widely recognized 23. However, PD-1/PD-L1 blockade still has response rates below 40% in most cancer types 24 and is also limited by the lack of known biomarkers, immune-related toxicity, as well as innate and acquired drug resistance. Combination therapies are expected to address these limitations 23, but the occurrence of related serious adverse events (AEs) needs to be mitigated 25. Finding new drug combinations is currently one of the most promising antitumor approaches. We proposed an anti-tumor strategy of combining anti-PD-1 therapy with VD and validated the anti-cancer effect of VD-boosted PD-1 blockade on a patient-derived xenograft (PDX) model. Our findings provide a new option for developing more effective and less toxic anti-PD-1 immunotherapies.

## Results

### VDBP expression is correlated with HCC prognosis and VM

**Figure 1:**
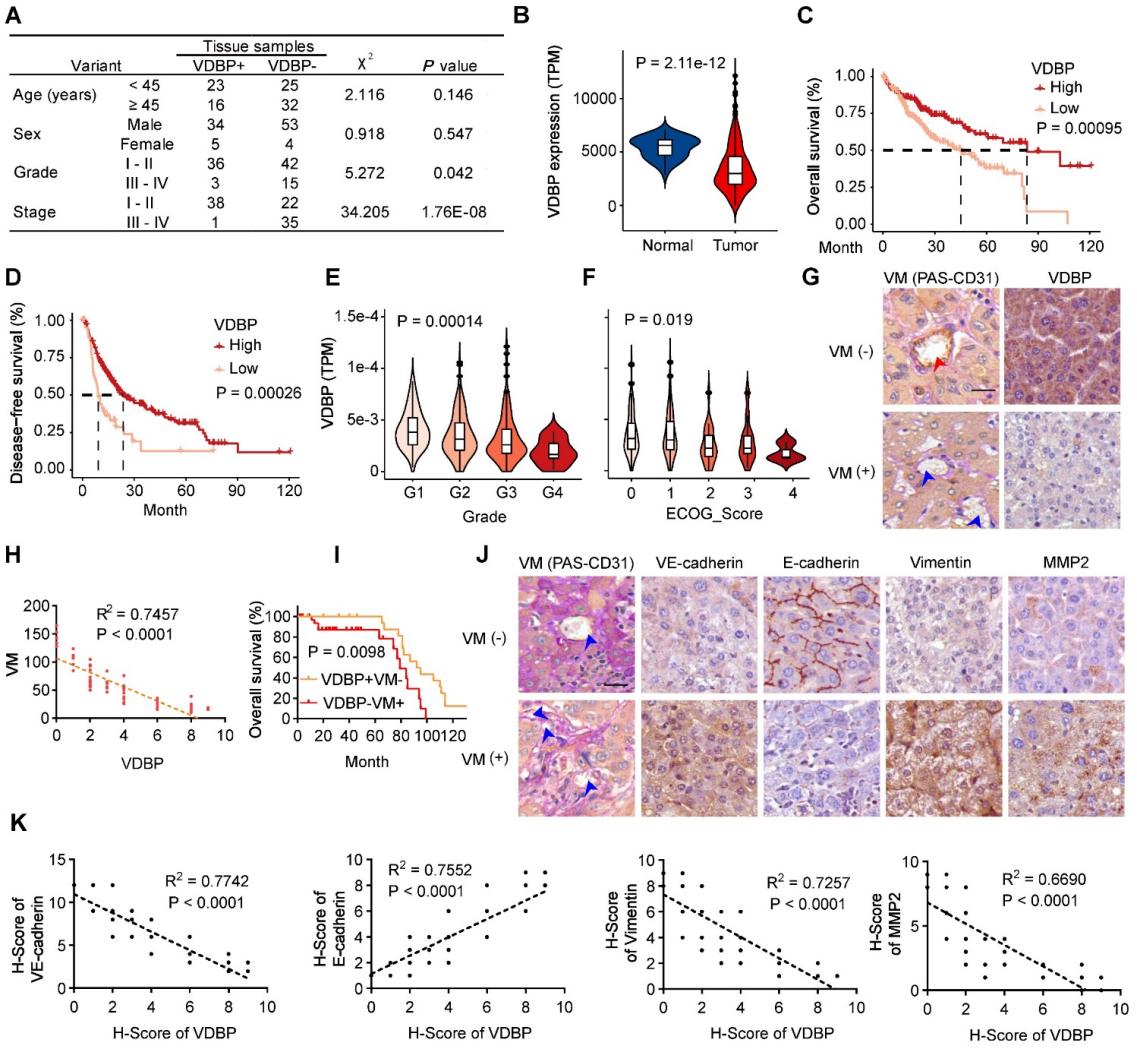
**VDBP expression is correlated with HCC prognosis and VM. (A)** Correlation between VDBP expression and clinicopathologic characteristics of patients with 95 case of HCC patients by chi-square test. **(B)** mRNA expression level (TPM) of VDBP in normal and tumor tissues of HCC in the TCGA dataset. **(C and D)** Kaplan-Meier curves showing the percentage of the overall survival (C) and disease-free survival (D) with higher and lower expression of VDBP in HCC from the TCGA dataset. **(E)** VDBP mRNA expression level (TPM) with different Grade (G1-G4) in the TCGA dataset. One-way ANOVA and Tukey's multiple comparisons test was used **(F)** VDBP mRNA expression level (TPM) with different ECOG score (0-4) in the TCGA dataset. One-way ANOVA and Tukey's multiple comparisons test was used. **(G)** IHC staining of VDBP expression in VM (-) (the number of VMs less than 10) and VM (+) (the number of VMs equal to or more than 10) HCC samples. Representative images of CD31-PAS co-staining and VDBP IHC staining are shown on the left and right respectively. The location of the endothelial-dependent vessels (both positive for CD31 and PAS) was indicated with a red arrow and the location of the VM (positive for PAS and negative for CD31) was indicated with a blue arrow. Scale bar, 40 µm. **(H)** Correlation analysis of VDBP and VM. (n = 75). Statistics were calculated on biological replicates with simple linear regression. **(I)** Kaplan-Meier curve of the overall survival rate of HCC patients with VDBP/VM (+/-: n = 23; -/+: n = 39). **(J)** Representative images of CD31-PAS co-staining and IHC staining of VE-cadherin, E-cadherin, Vimentin, MMP2 proteins in VM (-) and VM (+) HCC samples. The location of the VM (positive for PAS and negative for CD31) was indicated with a blue arrow. Scale bar,40 µm. **(K)** Correlation analysis of VDBP with VE-cadherin, E-cadherin, Vimentin, and MMP2, respectively. n = 75. Statistics were calculated on biological replicates with simple linear regression.

To investigate the role of VDBP in HCC, we first analyzed the correlation between VDBP expression and clinical grade and stage by IHC staining in tumor tissues of 96 HCC patients, as shown in Figure 1A, VDBP was lowly expressed in advanced grades and stages and significantly elevated in the early stage of tumorigenesis. To obtain more clinical information about VDBP, we further analyzed the correlation between VDBP expression and HCC in the TCGA database, consistently, the mRNA expression of VDBP was significantly higher in adjacent normal tissue compared with tumor tissue (Figure 1B). Furthermore, high VDBP expression was positively associated with longer overall and disease-free survival (Figure 1C-D), as well as with earlier clinicopathological grading (Figure 1E), and lower ECOG scores (Figure 1F) in patients with HCC. Next, we classified the tissues of 75 HCC patients into VM (+) and VM (-) groups by CD31-PAS double staining based on the number of VMs in the pathological tissues. Together with the IHC staining analysis of VDBP, we found that the VDBP expression was negatively correlated with the number of VMs (Figure 1G-H). We then further divided the patients into VDBP (-) VM (+) and VDBP (+) VM (-) groups, and survival analysis suggested that patients in the VDBP (+) VM (-) group had a significantly longer survival (Figure 1I). Subsequently, we performed IHC staining analysis of VM-related markers, including VM markers VE-cadherin, Fibronectin 1 (FN1), SERPINE2, tumor microenvironment markers MMP2 and MMP9, epithelial cell marker E-cadherin and mesenchymal cell marker vimentin (Figure 1J-K and Figure S1A-B). The co-expression analysis showed that VDBP expression was negatively correlated with the expression of the VM, tumor microenvironment, and mesenchymal cell markers, whereas it was positively correlated with the epithelial marker. Collectively, the above results suggest that a high VDBP expression is closely associated with a favorable clinical prognosis of HCC and that VDBP is involved in regulating the formation of VM.

### VM is regulated by VDBP in HCC

**Figure 2:**
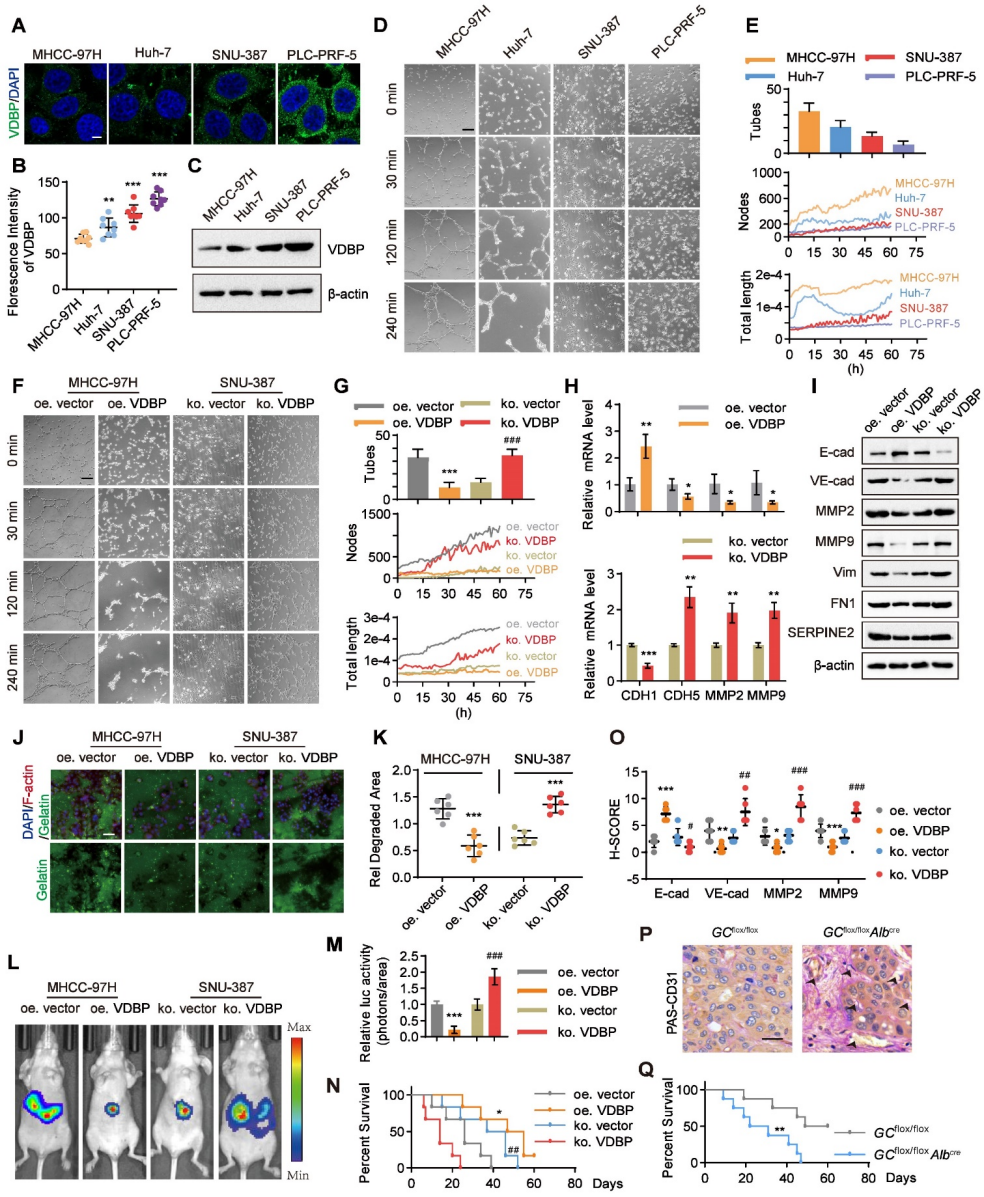
**VM is regulated by VDBP in HCC. (A-B)** Representative images (A) and quantification of florescence intensity (B) of VDBP immunofluorescence staining in MHCC-97H, Huh-7, SNU-387 and PLC-PRF-5 cells. Scale bar, 5 µm. n = 8, biological replicates. Statistics were calculated on biological replicates with two-tailed unpaired *t*-tests, **P < 0.01, ***P < 0.001, compared with the MHCC-97H group. Error bars show mean with SD. **(C)** Western blot analysis of VDBP expression level in MHCC-97H, Huh-7, SNU-387 and PLC-PRF-5 cells. **(D and E)** Matrigel 3D culture as an *in vitro* model to study VM formation. Representative plots of VM status of MHCC-97H, HUH-7, SNU-387 and PLC-PRF-5 cells at 0, 30, 120 and 240 min (D) and quantitative plots of the three measured parameters for the four groups of cells (E). The median time point of live cell imaging (30 h) was firstly selected for traditional VM statistics (Tubes), and VM-related parameters (Nodes and Total length) variation curves for 60h were analyzed by AngiogenesisAnalyzer.ijm, AutomaticMeasure.ijm, and VM.R codes. Scale bar, 200 µm. **(F and G)** Representative plots of VM status of MHCC-97H and SNU-387 treated as indicated at 0, 30, 120 and 240 min (F) and quantitative plots of the three measured parameters for the four groups of cells (G). Scale bar, 200 µm. ***P < 0.001, compared with the oe vector group.^ ###^*P* < 0.001, compared with the ko vector group. **(H)** The levels of CDH1, CDH5, MMP2 and MMP9 mRNA were detected by RT-qPCR in MHCC-97H (top) and SNU-387 cells (bottom) with Matrigel 3D culture treated as indicated. n = 3, biological replicates. Statistics were calculated on biological replicates with two-tailed unpaired *t*-tests, *P < 0.05, **P < 0.01, ***P < 0.001, compared with the oe vector group (top), compared with the ko vector group (bottom). Error bars show mean with SD.** (I)** The levels of VM related proteins were detected by western blot in MHCC-97H and SNU-387 cells with Matrigel 3D culture treated as indicated. **(J and K)** Fluorescent gelatin degradation and phalloidin/DAPI staining of MHCC-97H and SNU-387 cells treated as indicated (J) and quantification of degradation area (K). Scale bar, 50 µm. n = 6, biological replicates. Statistics were calculated on biological replicates with two-tailed unpaired *t*-tests, ***P < 0.001, compared with the vector group. Error bars show mean with SD.** (L and M)** Representative in vivo images (L) of MHCC-97H/SNU-387-luc-tumor-bearing-BALB/c-nude mice with indicated treatments and quantified values (M) are shown as relative luciferase activity (photons/area). n = 6, biological replicates. Statistics were calculated on biological replicates with two-tailed unpaired *t*-tests, ***P < 0.001, compared with the oe.vector group.^ ###^*P* < 0.001, compared with the ko vector group. Error bars show mean with SD. **(N)** Kaplan-Meier curves showing percentage of survival of MHCC-97H/SNU-387-luc-tumor-bearing-BALB/c-nude mice after indicated treatments. *P < 0.05, compared with the oe vector group.^ ##^*P* < 0.01, compared with the ko vector group. **(O)** Quantification of pathological score of IHC staining of E-cadherin, VE-cadherin, MMP2 and MMP9 in liver tissues of MHCC-97H/SNU-387-luc-tumor-bearing-BALB/c-nude mice. Scale bar, 40 µm. n = 6, biological replicates. Statistics were calculated on biological replicates with two-tailed unpaired *t*-tests, *P < 0.05, **P < 0.01, ***P < 0.001, compared with the oe vector group.^ #^*P* < 0.05,^ ##^*P* < 0.01, ^###^*P* < 0.001, compared with the ko vector group. Error bars show mean with SD.** (P)** Representative images of PAS-CD31 co-staining on liver tissues of mice created using Cre-lox technology with a hepatocyte-specific *GC* deletion by backcrossing *GC*^flox/flox^ mice to Alb-Cre mice. Scale bar, 40 µm.** (Q)** Kaplan-Meier curves showing percentage of survival of *GC*^flox/flox^ mice and *GC*^flox/flox^*Alb*^cre^mice. **P < 0.01, compared with the *GC*^flox/flox^ group.

We aimed to further investigate the correlation between VDBP expression levels and the ability of VM formation in HCC cells. Initially, we examined the expression levels of VDBP in four HCC cell lines, including MHCC-97H, Huh-7, SNU-387, and PLC-PRF-5 through immunofluorescence (IF) staining. We found that the fluorescence intensity of VDBP was significantly stronger in SNU-387 and PLC-PRF-5 cells than in MHCC-97H and Huh-7 cells. Particularly, PLC-PRF-5 cells exhibited the strongest VDBP fluorescence intensity, whereas the weakest was observed in MHCC-97H cells (Figure 2A-B). The immunoblotting results of VDBP in the four cell lines were consistent with the IF results (Figure 2C). Subsequently, we evaluated the VM formation ability of these four cell lines using the classic three-dimensional cell culture model. We recorded the formation process of VM through live cell imaging and counted the number of VMs formed by each cell line using the established VM evaluation method (Figure 2D-E), and also drew the change curves of VM-related features including node and total length for 60 h to further evaluate the VM formation ability based on our new established methodology 26 (Figure 2E). The results showed that MHCC-97H cells formed the most VMs; the VM-related features, number of nodes, and total length continuously increased within 60 h. Huh-7 cells formed fewer VMs than MHCC-97H cells, and the number of nodes increased slowly within 60 h, while the total length reached its peak at 14 h and then sharply decreased. SNU-387 cells formed fewer VMs, and their VM-related features increased slowly within 60 h. PLC-PRF-5 cells formed the least VMs, and neither the number of nodes nor total length showed an increasing trend. Our results suggested that MHCC-97H cells have the strongest ability to form VMs, while SNU-387 cells are less likely to form VMs, and PLC-PRF-5 cells have the weakest ability to form VMs. This was consistent with the expression level of VDBP in these four cell lines, indicating that cell lines with low expression of VDBP have a stronger ability to form VMs. To further investigate the regulation of VM by VDBP in cells, we overexpressed VDBP in the MHCC-97H cell line, which has low VDBP expression and strong VM-forming ability, and knocked out VDBP in SNU-387 cells, which have high VDBP expression and weak VM-forming ability, to evaluate their ability to form VM (Figure 2F-G). The results showed that overexpression of VDBP significantly reduces the ability of MHCC-97H to form VM, while knocking out VDBP remarkably enhances the ability of SNU-387 to form VM. qPCR results also showed a significant downregulation of VM-related markers, including CDH5, MMP2, and MMP9, and a significant upregulation of the epithelial cell marker E-cadherin in cells overexpressing VDBP. Conversely, knocking out VDBP resulted in a remarkable increase in VM marker expression and a notable decrease in E-cadherin expression (Figure 2H). Consistently, Western blot analysis showed similar results (Figure 2I), indicating that VDBP inhibits the formation of VM in cells.

Given that VM is a manifestation of tumor cell evolution and EMT 27, we sought to examine the potential of VDBP to impede migration and invasion of HCC cells. To this end, we conducted fluorescent gelatin degradation assays on MHCC-97H and SNU-387 cells, wherein VDBP was either overexpressed or knocked out, as shown in Figure 2J-K. Our findings indicated that overexpression of VDBP significantly curtails the migration and invasion capacity of HCC cells in degrading gelatin, whereas VDBP knockout promotes these behaviors. To further investigate the matter, we established an *in vivo* model of liver orthotopic transplantation. The results suggested that overexpression of VDBP effectively suppresses tumor growth and considerably extends the survival of mice. Conversely, the knockout of VDBP resulted in the promotion of tumor growth and a shorter survival time in mice (Figure 2L-N). The IHC staining results of mouse tumor tissues further revealed a noteworthy decrease in VM-related markers and a significant increase in the epithelial marker E-cadherin following VDBP overexpression, with the opposite trend observed after VDBP knockout (Figure 2O and Figure S2). Based on the hepatocyte-specific *GC* deletion mouse model, we found more VMs in the liver of the *GC* knockout mice group than in the control group (Figure 2P). Furthermore, a significantly shorter survival was observed in mice with *GC* deletion (Figure 2Q). In conclusion, VDBP exhibited the potential to impede VM, thereby inhibiting the progression of HCC *in vitro* and *in vivo*.

### VDBP interacts with Twist1 to inhibit Twist1-activated VE-cadherin transcription

**Figure 3:**
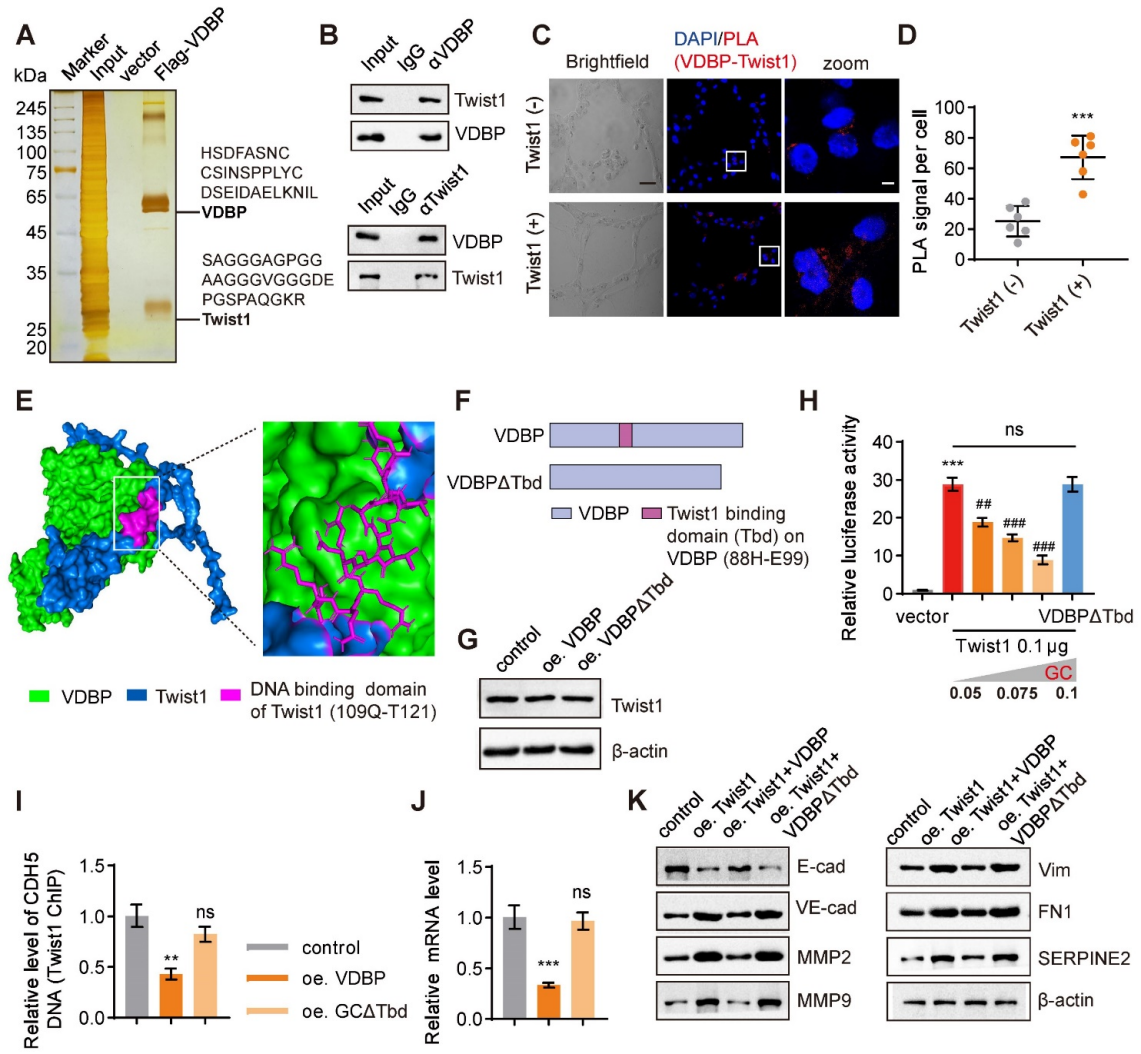
**GC interacts with Twist1 to inhibit Twist1-activated VE-cadherin transcription. (A)** Silver staining of proteins acquired by FLAG-VDBP pull-down in cell lysis from MHCC-97H cells with Matrigel 3D culture. **(B)** Co-immunoprecipitation assays in MHCC-97H cells on Matrigel 3D culture with anti-VDBP followed by immunoblotting (IB) with antibodies against Twist1 and VDBP or with anti-Twist1 followed by IB with anti-Twist1 and VDBP. **(C)** Brightfield pictures (left) and fluorescent pictures of PLA assays with VDBP and Twist1 (right) in Twist1-negative cell (SNU-387) and Twist1-positive cell (SK-HEP-1) with Matrigel 3D culture. Scale bar, 40 µm for black and 5 µm for white. **(D)** Quantification graph of PLA signal per cell. n = 6, biological replicates. Statistics were calculated on biological replicates with two-tailed unpaired *t*-tests, ***P < 0.001. Error bars show mean with SD. **(E)** Visualization result of simulation docking between VDBP and Twist1. **(F)** Schematic diagram of full-length and truncated VDBP proteins. **(G)** Western blot analysis of Twist1 expression level in MHCC-97H cells with Matrigel 3D culture treated as indicated. **(H)** The transcriptional regulation of VE-cadherin by transfection of Twist1 and increasing VDBP plasmids detected with the dual-luciferase reporter assay. n = 6, biological replicates. Statistics were calculated on biological replicates with two-tailed unpaired *t*-tests, ***P < 0.001, compared with vector group, ^##^*P* < 0.01, ^###^*P* < 0.001, compared with the transfection of Twist1 alone group. Error bars show mean with SD.** (I)** Results of qPCR after Twist1 ChIP of CDH5 target gene in MHCC-97H cells with Matrigel 3D culture treated as indicated. n = 3, biological replicates. Statistics were calculated on biological replicates with two-tailed unpaired *t*-tests, **P < 0.01, ns, not significant, compared with the control group. Error bars show mean with SD. **(J)** The levels of CDH5 mRNA were detected by RT-qPCR in MHCC-97H cells with Matrigel 3D culture treated as indicated. n = 3, biological replicates. Statistics were calculated on biological replicates with two-tailed unpaired *t*-tests, ***P < 0.001, ns, not significant, compared with the control group. Error bars show mean with SD. **(K)** The levels of VM related proteins were detected by western blot in MHCC-97H cells with Matrigel 3D culture treated as indicated.

We further used a three-dimensional cell culture model of MHCC-97H cells with overexpression of VDBP to investigate the regulation of VM by VDBP in HCC. Through a pull-down experiment, in conjunction with MS analysis, we identified Twist1, a crucial transcription factor that promotes both VM and EMT processes, as the protein that interacts with VDBP (Figure 3A).

Previous studies have indicated that Twist1 can directly bind to the promoter region of VE-cadherin and activate its expression, hereby promoting VM formation 17. The interaction between VDBP and Twist1 was further verified by a Co-IP experiment (Figure 3B). Subsequently, we detected the interaction between VDBP and Twist1 in cells exhibiting varying levels of Twist1 expression using a proximity ligation assay (PLA). Our findings indicated a significant increase in the interaction between VDBP and Twist1 in cells with high Twist1 expression (Figure 3C-D). The interaction sites between VDBP and Twist1 were found by computer simulation docking technology; VDBP exhibited binding affinity towards the main domain responsible for DNA binding of Twist1 (^109^Q-T^121^) 28 (Figure 3E). It was thus reasoned that the VDBP-Twist1 interaction may have an impact on the gene-regulatory function of Twist1. To investigate the effect of the interaction between VDBP and Twist1, we deleted the ^88^H-E^99^ fragment on VDBP that interacts with the Twist1 DNA binding domain and constructed VDBPΔTbd as a negative control (Figure 3F). Subsequently, we overexpressed VDBP or VDBPΔTbd in a three-dimensional cell culture of cells with high expression of Twist1 and detected the changes in Twist1 content through western blot, and found that neither overexpression of VDBP nor VDBPΔTbd affected the expression of Twist1 (Figure 3G). We further studied the role of VDBP in the Twist1-mediated expression of VE-cadherin. As shown in Figure 3H, the presence of VDBP seemed to inhibit the transcription of VE-cadherin, while VDBPΔTbd did not seem to have any repressive influence on Twist1-induced CDH5 luciferase expression. Furthermore, CHIP-qPCR results demonstrated that VDBP inhibited Twist1 binding to the CDH5 promoter (Figure 3I) and suppressed the mRNA expression level of VE-cadherin (Figure 3J). The expression of VM-related markers was upregulated by Twist1, while VDBP overexpression restored this process (Figure 3K). These results suggest that VDBP hijacks Twist1 upon interaction and inhibits the transcriptional activation of Twist1-induced VE-cadherin.

### VDBP hijacks Twist1 to inhibit VM and suppresses HCC progression

**Figure 4:**
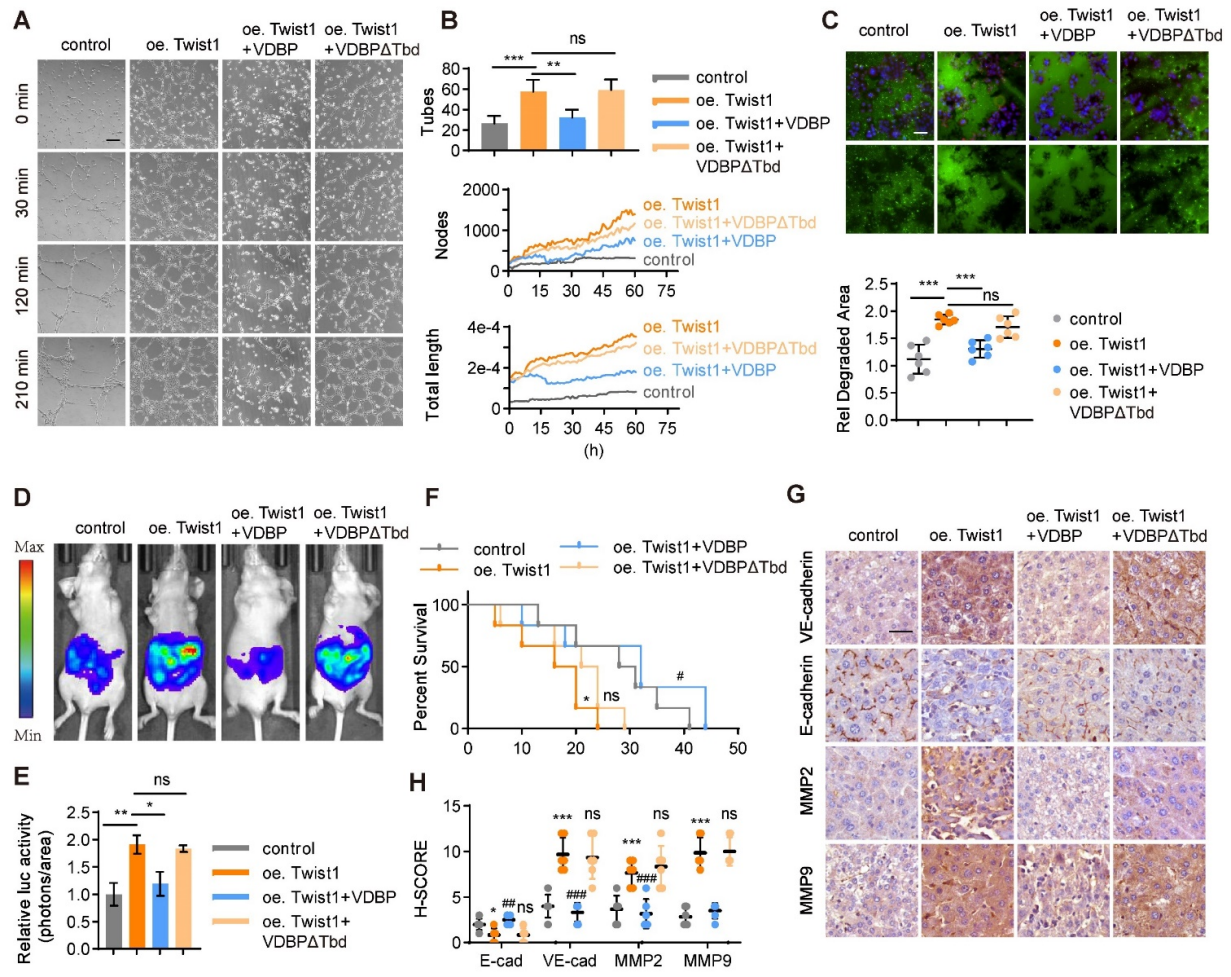
** VDBP hijacks Twist1 to inhibit VM and suppresses the malignancy of HCC, supplementing VDBP can weaken the promoting effect of Twist1 on VM and malignant progression in HCC. (A and B)** Representative plots of VM status of MHCC-97H cells at 0, 30, 120 and 240 min (A) and quantitative plots of the two measured parameters (B). Scale bar, 200 µm. **(C)** Fluorescent gelatin degradation and phalloidin/DAPI staining of MHCC-97H cells treated as indicated (top) and quantification of degradation area (bottom). Scale bar, 50 µm. n = 6, biological replicates. Statistics were calculated on biological replicates with two-tailed unpaired *t*-tests, ***P < 0.001, ns, not significant. Error bars show mean with SD. **(D and E)** Representative *in vivo* images (D) of MHCC-97H-luc-tumor-bearing-BALB/c-nude mice with indicated treatments and quantified values (E) are shown as relative luciferase activity (photons/area). n = 6, biological replicates. Statistics were calculated on biological replicates with two-tailed unpaired *t*-tests, *P < 0.05, **P < 0.01, ns, not significant. Error bars show mean with SD. **(F)** Kaplan-Meier curves showing percentage of survival of MHCC-97H-luc-tumor-bearing-BALB/c-nude mice after indicated treatments.** (G and H)** Representative images (G) and quantification of pathological score (H) of IHC staining of VE-cadherin, E-cadherin, MMP2 and MMP9 in liver tissues of MHCC-97H-luc-tumor-bearing-BALB/c-nude mice. Scale bar, 40 µm. n = 6, biological replicates. Statistics were calculated on biological replicates with two-tailed unpaired *t*-tests, *P < 0.05, ***P < 0.001, compared with Control. ^##^*P* < 0.01, ^###^*P* < 0.001, ns, not significant. compared with oe Twist1. Error bars show mean with SD.

A three-dimensional cell culture of MHCC-97H was conducted to study the impact of VDBP hijacking Twist1 on the function of HCC. Our findings indicated that Twist1 overexpression enhances VM formation and that VDBP supplementation could reverse this process (Figure 4A-B). Additionally, we found that VDBP inhibited Twist1-induced migration and invasion in HCC cells (Figure 4C). Furthermore, we established an *in vivo* liver orthotopic transplantation model, which showed that Twist1 overexpression promotes tumor growth in mice, and VDBP supplementation significantly inhibited the Twist1-induced effects and prolonged the survival of mice (Figure 4D-F). Moreover, IHC staining analysis of mouse tumor tissues revealed that VDBP inhibits the expression of VM-related markers induced by Twist1 (Figure 4G-H). These results suggest that VDBP inhibits the formation of VM by hijacking Twist1, thereby suppressing the malignant progression of HCC.

### VD promotes VDBP expression dependent on VDR

**Figure 5:**
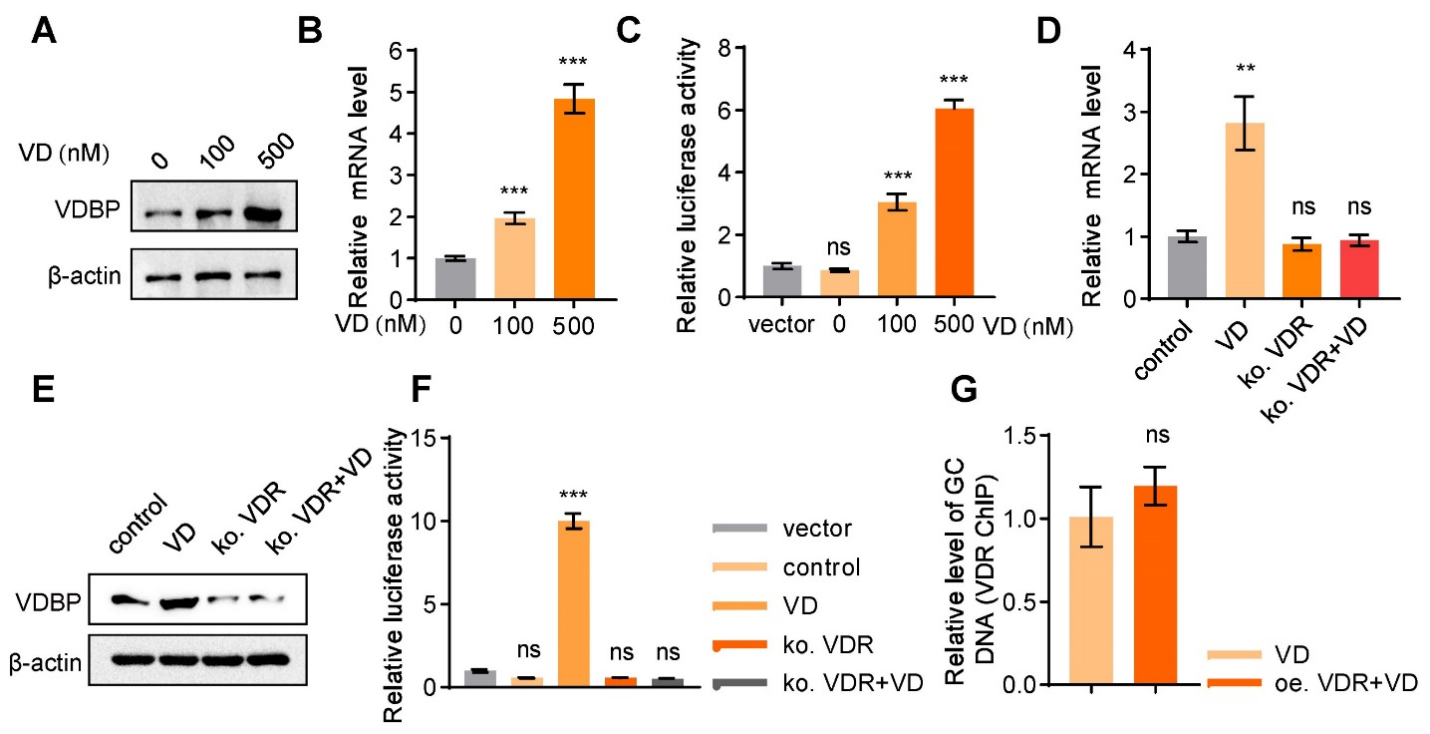
**VD promotes VDBP expression dependent on VDR. (A)** Western blot analysis of VDBP protein expression level in MHCC-97H cells with Matrigel 3D culture treated with 1,25-hydroxy vitamin D3 for 48 h.** (B)** The levels of VDBP mRNA were detected by RT-qPCR in MHCC-97H cells with Matrigel 3D culture treated as indicated for 48 h. n = 3, biological replicates. Statistics were calculated on biological replicates with two-tailed unpaired *t*-tests, ***P < 0.001, compared with Control. Error bars show mean with SD. **(C)** The transcriptional regulation of VDBP by different concentrations of VD treatment for 48 h detected through the dual-luciferase reporter assay. n = 3, biological replicates. Statistics were calculated on biological replicates with two-tailed unpaired *t*-tests, ***P < 0.001, ns, not significant. Error bars show mean with SD.** (D)** The levels of VDBP mRNA were detected by RT-qPCR in MHCC-97H cells with Matrigel 3D culture treated as indicated for 48 h. n = 3, biological replicates. Statistics were calculated on biological replicates with two-tailed unpaired *t*-tests, **P < 0.01, ns, not significant. Error bars show mean with SD.** (E)** Western blot analysis of VDBP protein expression level in MHCC-97H cells with Matrigel 3D culture treated as indicated for 48 h.** (F)** The transcriptional regulation of VDBP by different treatments for 48 h detected through the dual-luciferase reporter assay. n = 3, biological replicates. Statistics were calculated on biological replicates with two-tailed unpaired *t*-tests, ***P < 0.001, ns, not significant. Error bars show mean with SD.** (G)** Results of qPCR after VDR ChIP of VDBP target gene in MHCC-97H cells with Matrigel 3D culture treated as indicated for 48 h. n = 3, biological replicates. Statistics were calculated on biological replicates with two-tailed unpaired *t*-tests, ns, not significant. Error bars show mean with SD.

The demonstrated role of VDBP in suppressing HCC prompted further investigation into the regulatory mechanisms of VDBP expression. Treatment with VD resulted in a dose-dependent increase in VDBP protein expression levels in MHCC-97H cells (Figure 5A), which was further supported by consistent findings from the detection of VDBP mRNA expression levels (Figure 5B). Additionally, the activation of *GC* transcription by VD was observed (Figure 5C). It is well-known that VD functions through the VDR, whereby VD promotes the nuclear translocation of the VDR, which forms a heterodimer with retinoid X receptor (RXR) and binds to the promoter region of target genes to regulate their expressions 29. Our study revealed that after VDR knockout in MHCC-97H cells, VD has no longer an effect on the protein or mRNA expression levels of VDBP (Figure 5D-E), nor could it activate the transcription of *GC* (Figure 5F), suggesting that VD exerts its regulatory effects on VDBP through the VDR. As a transcription factor, VDR is capable of binding to vitamin D response elements (VDREs) located in gene promoter regions, thereby regulating gene expression 30. Nevertheless, the absence of VDREs in the *GC* promoter region precludes VDR from directly regulating VDBP expression. Moreover, we found that overexpression of VDR could not further promote the binding of VDR to the *GC* promoter in the presence of VD (Figure 5G), which implied that the VDR may not be a direct factor in VDBP transcriptional activation.

### VDR interacts with YY1 to activate the transcription of VDBP

**Figure 6:**
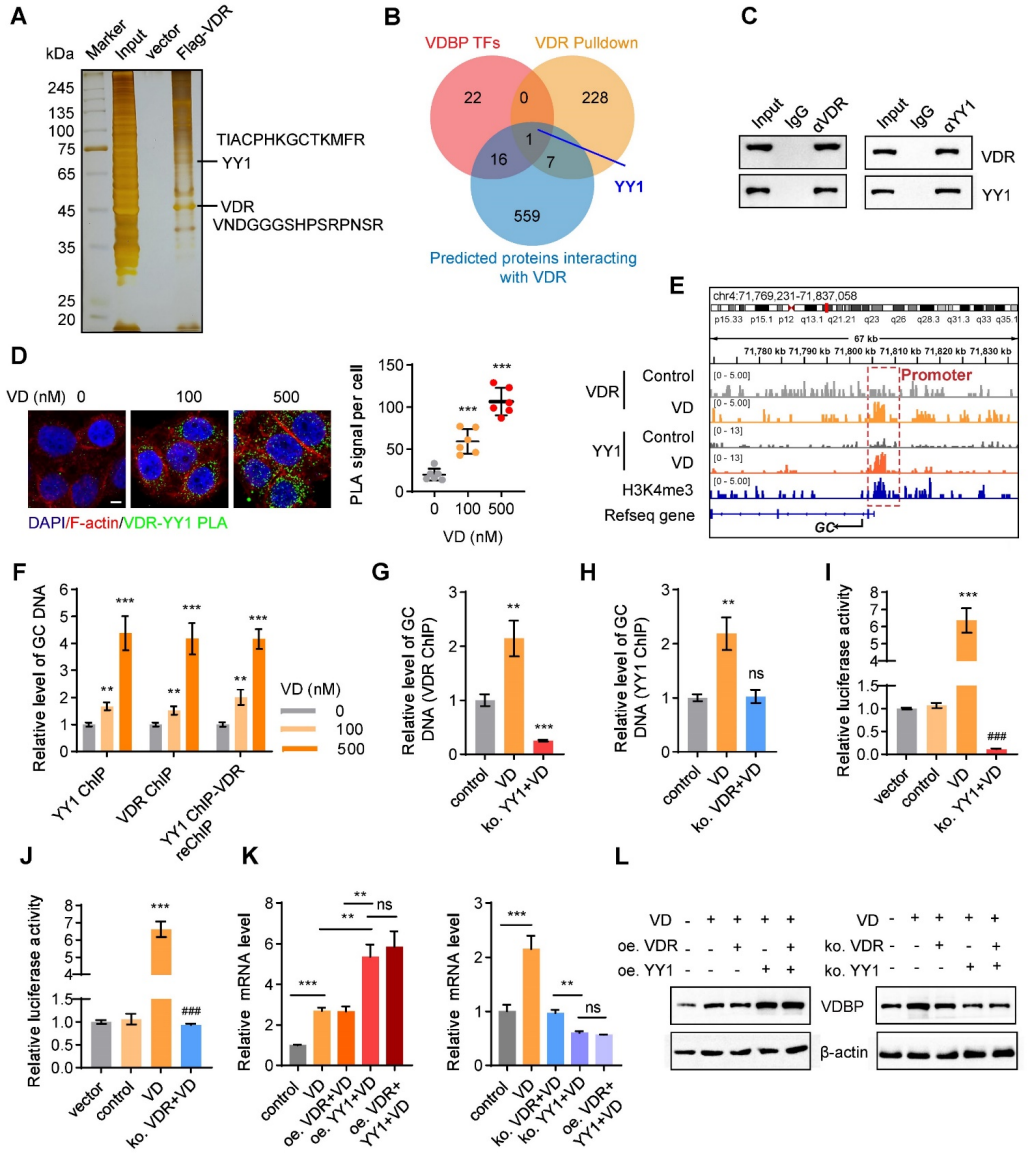
**VDR interacts with YY1 to activate the transcription of VDBP. (A)** Silver staining of proteins acquired by Flag-VDR pull-down in nuclear lysis from MHCC-97H cells. **(B)** Venn diagram for screening YY1. **(C)** Co-immunoprecipitation assays in MHCC-97H cells with anti-VDR followed by immunoblotting (IB) with antibodies against VDR and YY1 or with anti-YY1 followed by IB with anti- VDR and YY1. **(D)** Fluorescent pictures (left) and quantification graph (right) of PLA assays with VDR and YY1 in MHCC-97H cells with Matrigel 3D culture with different concentrations of VD. Scale bar, 5 µm. Quantification graph of PLA signal per cell. n = 6, biological replicates. Statistics were calculated on biological replicates with two-tailed unpaired t-tests, ***P < 0.001, compared with the 0 group. Error bars show mean with SD. **(E)** ChIP-seq peaks at the *GC* gene after VD treatment in MHCC-97H cells. **(F)** Results of qPCR after YY1 ChIP / VDR ChIP / YY1 ChIP - VDR re-ChIP of *GC* target gene in MHCC-97H cells treated as indicated. n = 3, biological replicates. Statistics were calculated on biological replicates with two-tailed unpaired t-tests, **P < 0.01, ***P < 0.001, compared with the 0 group. Error bars show mean with SD. **(G)** Results of qPCR after VDR ChIP of *GC* target gene in MHCC-97H cells treated as indicated. n = 3, biological replicates. Statistics were calculated on biological replicates with two-tailed unpaired t-tests, **P < 0.01, ***P < 0.001, compared with the control group. Error bars show mean with SD. **(H)** Results of qPCR after YY1 ChIP of *GC* target gene in MHCC-97H cells treated as indicated. n = 3, biological replicates. Statistics were calculated on biological replicates with two-tailed unpaired *t*-tests, **P < 0.01, ns, not significant, compared with the 0 group. Error bars show mean with SD. **(I and J)** Detection of transcriptional activation of the *GC* by different treatment conditions using the dual luciferase reporter assay. n = 3, biological replicates. Statistics were calculated on biological replicates with two-tailed unpaired *t*-tests, ***P < 0.001, compared with the control group. ^###^*P* < 0.001, compared with the VD group. Error bars show mean with SD. **(K)** The levels of VDBP mRNA were detected by RT-qPCR in MHCC-97H cells treated as indicated.** (L)** Western blot analysis of VDBP expression level in MHCC-97H cells treated as indicated. n = 3, biological replicates. Statistics were calculated on biological replicates with two-tailed unpaired *t*-tests, **P < 0.01, ***P < 0.001, ns, not significant. Error bars show mean with SD.

To explore the relevant proteins of the VDR in MHCC-97H cells, we performed pull-down assays followed by MS analysis (Figure 6A). Upon conducting a Venn analysis on the predicted VDBP transcription factors from the *Cistrome* database, the VDR interacting proteins identified by MS, and the VDR interacting proteins in the FPClass database, the presence of Yin-Yang 1 (YY1) was found (Figure 6B). YY1, belonging to the GLI-Kruppel family of transcription factors, acts as a DNA binding protein and is involved in numerous biological processes, including cell growth, embryonic development, transcriptional regulation, and tumorigenesis 31, 32. The interaction between the VDR and YY1 was validated through Co-IP and PLA experiments, and an increase in their interaction was observed with an increase in VD dosage (Figure 6C-D). Subsequently, we studied the transcriptional regulation effect of the VDR and YY1 in MHCC-97H cells using CHIP-seq, under DMSO or VD treatment. The results revealed the presence of binding sites upstream of the *GC* transcriptional starting site (TSS). Enriched YY1 and VDR peaks are displayed in Figure 6E. Notably, following VD treatment, there was a significant increase in YY1 and VDR peak enrichment on the GC promoter, indicating that VD promotes the regulation of VDBP by VDR and YY1. CHIP-qPCR analysis was carried out on MHCC-97H cells treated with different concentrations of VD by using specific antibodies against YY1 or VDR, and the occupancy of YY1 and VDR on the *GC* promoter was observed which validated the CHIP-seq results (Figure 6F). To study whether YY1 directly binds to the promoter region of *GC*, we separately knocked out YY1 and VDR in MHCC-97H cells before treatment with VD. CHIP-qPCR was conducted using VDR antibodies in YY1 knockout cells and using YY1 antibodies in VDR knockout cells. YY1 knockout made VD ineffective in promoting the recognition of VDR on the *GC* promoter which resulted in a significant reduction in its recognition (Figure 6G), and upon VDR knockout, VD was observed to be unsuccessful in promoting the binding of YY1 to the *GC* promoter, while not affecting its baseline binding level (Figure 6H). These results indicated that YY1 directly binds to the promoter region of *GC*. To assess the impact of YY1 and VDR on VDBP transcription, we performed a dual-luciferase reporter assay. MHCC-97H cells were transfected with the PGL3-promoter plasmid, which includes the YY1 and VDR binding motifs, either alone or co-transfected with the pcDNA3.1-3× Flag-YY1 plasmid. The results indicated that the activation of transcription of *GC* by YY1 is facilitated by VD through the VDR (Figure 6I-J). Subsequently, overexpression and knockout of VDR and YY1 were respectively performed in MHCC-97H and SNU-387 cells, and qPCR and western blot analyses were carried out to detect changes in the expression levels of VDBP. It was revealed that in the presence of VD, YY1 overexpression facilitated the transcription and translation of VDBP, whereas the expression levels of VDR did not. Following YY1 knockout, VDBP expression was not enhanced by VD, and simultaneous knockout of VDR and YY1 resulted in VDBP expression levels comparable to those observed in the YY1 knockout group (Figure 6K-L). In conclusion, VD promotes YY1-mediated transcriptional activation of VDBP by promoting the interaction between VDR and YY1.

### VD potentiates the anti-tumor effect of anti-PD-1 in HCC

**Figure 7:**
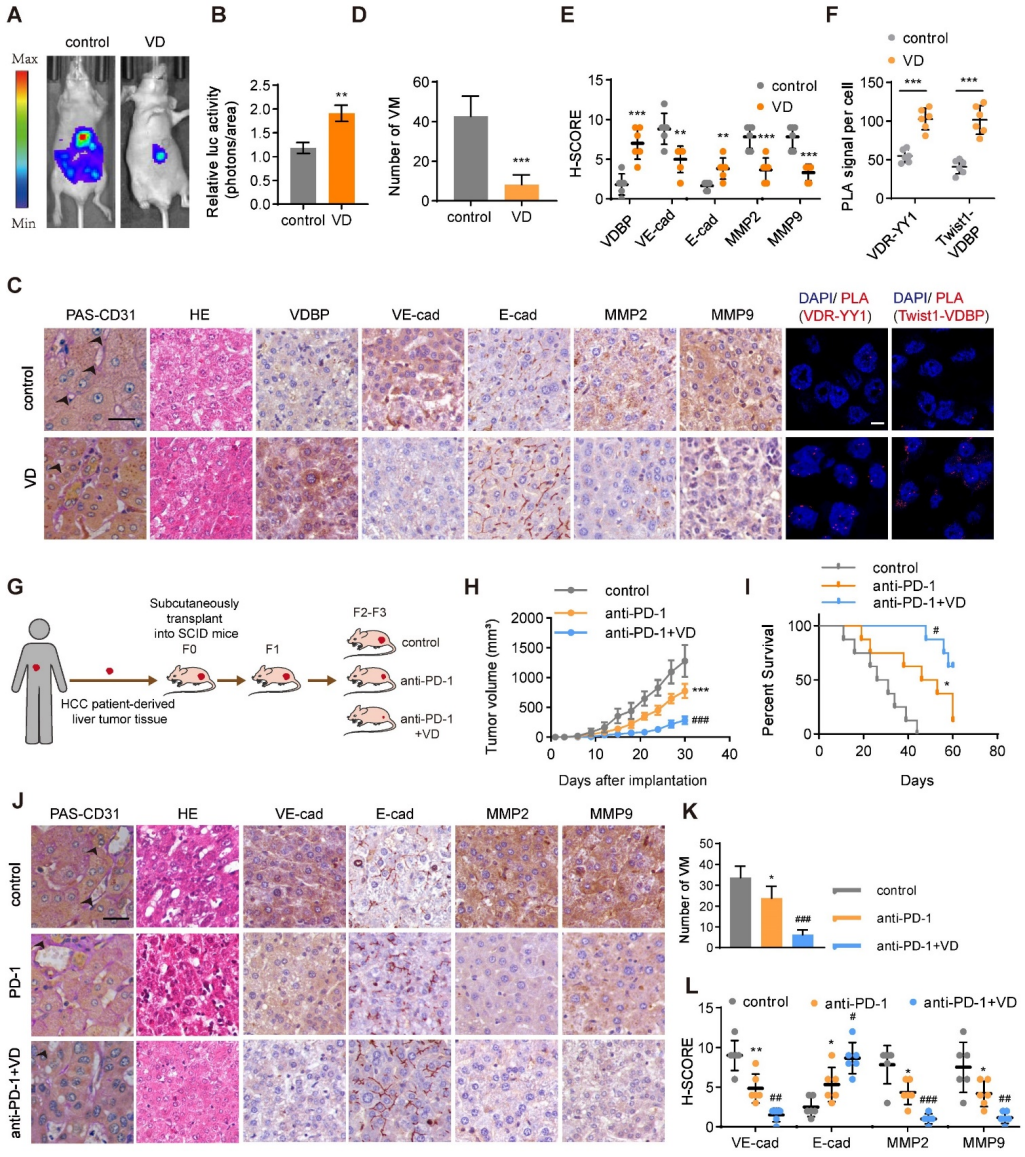
**VD potentiates the antitumor effect of anti-PD-1 in HCC. (A and B)** Representative *in vivo* images (A) of MHCC-97H-luc-tumor-bearing-BALB/c-nude mice with indicated treatments and quantified values (B) are shown as relative luciferase activity (photons/area). n = 6, biological replicates. Statistics were calculated on biological replicates with two-tailed unpaired *t*-tests, **P < 0.01. Error bars show mean with SD. **(C)** Representative images of CD31-PAS double staining, HE staining, IHC staining of VDBP, VE-cadherin, E-cadherin, MMP2 and MMP9 and PLA assays in liver tissues of MHCC-97H-luc-tumor-bearing-BALB/c-nude mice. Scale bar, 40 µm. **(D-F)** Quantification of number of VM (D), pathological score of IHC staining (E) and PLA signal per cell (F). n = 6, biological replicates. Statistics were calculated on biological replicates with two-tailed unpaired *t*-tests, **P < 0.01, ***P < 0.001. Error bars show mean with SD.** (G)** Flowchart on the construction of PDX model and antitumor test of anti-PD-1 or anti-PD-1/VD combination therapy *in vivo*. **(H)** The tumor volume was monitored every 3 days after implantation. n = 6, biological replicates. Statistics were calculated on biological replicates with two-tailed unpaired *t*-tests, ***P < 0.001, compared with Control, ^###^*P* < 0.001, compared with anti-PD-1. Error bars show mean with SD.** (I)** Kaplan-Meier curves showing percentage of survival of PDX model SCID mice after indicated treatments. n = 6, biological replicates. *P < 0.05, compared with Control, ^#^*P* < 0.05, compared with anti-PD-1. **(J)** Representative images of CD31-PAS double staining, HE staining and IHC staining of VE-cadherin, E-cadherin, MMP2 and MMP9 in liver tissues of PDX model SCID mice. Scale bar, 40 µm. **(K and L)** Quantification of number of VM (K) and pathological score of IHC staining (L)** in (J)**. n = 6, biological replicates. Statistics were calculated on biological replicates with two-tailed unpaired *t*-tests, *P < 0.05, **P < 0.01, compared with Control,^ #^*P* < 0.05,^ ##^*P* < 0.01, ^###^*P* < 0.001, compared with anti-PD-1. Error bars show mean with SD.

To evaluate the anti-tumor effect of VD, we established a liver orthotopic transplantation tumor model in BALb/c-nude mice. The imaging results demonstrated a significant reduction in tumor volume in mice supplemented with VD3 (Figure 7A-B).

We performed PAS-CD31 dual staining on tumor tissues from mice and subsequently counted the number of VMs; it was found that mice supplemented with VD3 had a significantly reduced number of VMs in their tumor tissues (Figure 7C-D). The results of HE staining demonstrated that mice receiving VD3 supplementation exhibited tumor cells characterized by uniform size, which suggested reduced heterogeneity (Figure 7C). Additionally, IHC staining suggested that VD3 supplementation suppressed the expression of VM-related markers while promoting the expression of E-cadherin (Figure 7C, E). PLA results on tumor tissue illustrated that the supplementation of VD3 promotes the interaction between YY1 and VDR at the tumor site, with a significant increase also observed in the interaction between VDBP and Twist1 (Figure 7C, F). The field of tumor therapeutics is increasingly focusing on immunotherapy, with anti-PD-1 being classical immune checkpoint inhibitors that are considered a promising strategy for tumor treatment. In this study, we evaluated the therapeutic effect of VD3 in combination with anti-PD-1 on tumors by establishing a PDX model (Figure 7G). We found that the combination therapy of VD and anti-PD-1 significantly reduced tumor volume, slowed tumor growth rate (Figure 7H), and prolonged survival of tumor-bearing mice (Figure 7I) compared with anti-PD-1 alone. Pathological analysis of tumor tissue sections showed that the number of VMs and VM-related marker expression was significantly reduced, while the expression of E-cadherin was increased in the drug combination treatment group (Figure 7J-L). These findings suggest that VD can enhance the anti-tumor effect and improve the efficacy of PD-1 inhibitors.

## Discussion

VDBP is a multifunctional protein whose primary functions, as indicated by current research, involve the binding and transport of vitamin D metabolites. The protein plays a crucial role in both physiological and pathological contexts, including immune and inflammatory regulation 33, 34, as well as serving as a biomarker for clinical diagnosis 35. Related studies have shown that there exists a significant correlation between the genetic polymorphism of VDBP and malignant tumors 2. However, the research on the mechanism for the role of VDBP in tumors is still not sufficiently comprehensive. Our findings have identified that VDBP expression plays a critical role in HCC; we identified that VDBP possesses the ability to suppress VM, consequently inhibiting the malignant progression of HCC. Since no prior work has shown the correlation of VDBP and VM, PAS-CD31 co-staining and IHC analysis of 75 patients with HCC were conducted. It was revealed that the expression of VDBP was negatively correlated with the quantity of VM and the expression levels of VM-related markers in HCC tumor tissues. Regarding the mechanism by which VDBP regulates VM, we discovered that VDBP can interact with the transcription factor Twist1 to obstruct recognition of Twist1 on the bHLH domain of genes, thereby preventing Twist1 from binding to the promoter region of VE-cad and thus inhibiting the expression of VE-cad, finally leading to the suppression of VM. Given that Twist1 is a key transcription factor in EMT, the interaction between VDBP and Twist1 can also inhibit classic EMT phenotypes such as migration and invasion. The study reported the initial instance of VDBP potentially modulating epigenetics through direct interaction with the transcription factor, thereby revealing a hitherto unexplored function of VDBP.

Numerous clinical studies have demonstrated the potential of VD as a treatment option for tumors 29, 36, 37. In this study, we deepened our understanding of VD regulating HCC progression and enhancing the effectiveness of immune therapy in HCC, by elucidating the specific molecular mechanism by which VD exerts its anti-tumor effect in HCC; VD promotes nuclear translocation of VDR-YY1 interaction, leading to transcriptional activation of VDBP. Animal studies revealed promising therapeutic results in the treatment of HCC through the upregulation of VDBP induced by VD, and a combination of VD and anti-PD-1 achieved remarkable results in the treatment of HCC.

## Supplementary Material

Supplementary figures and methods.

## Figures and Tables

**Table 1:** Materials

Reagents			Company	CAT#
Dual-Lumi™ Luciferase Reporter Gene Assay Kit			Beyotime	CAT#RG088s
Fast Silver Stain Kit			Beyotime	CAT#P0017S
CHIP Assay Kit			Beyotime	CAT#P2078
Hieff NGS® G-Type In-situ DNA Binding Profiling Library Prep Kit for Illumina®(BOXⅠ)			Yeasen	CAT#12598ES12
Hieff NGS® G-Type In-situ DNA Binding Profiling Library Prep Kit for Illumina®(BOXⅡ)			Yeasen	CAT#12598ES12
Color prestained protein molecular weight markers (10-245kD)			UE landy	CAT#P8028M
D-luciferin potassium>98%			meilunbio	CAT#115144-35-9
Duolink *In Situ* Detection Reagents Orange			Sigma Aldrich	CAT#DUO92007
Duolink *In Situ* PLA Probe Anti-Rabbit MINUS			Sigma Aldrich	CAT#DUO92005
Duolink *In Situ* PLA Probe Anti-Mouse PLUS			Sigma Aldrich	CAT#DUO92001
1,25-hydroxy vitamin D3			Med Chem Express	CAT#HY-10002
VD3			Med Chem Express	CAT#HY-15398
pork skin gelatin			Thermo Fisher	CAT#G13187
Matrigel® Matrix Basement Membrane HC Phenol-Red Free			CORNING	CAT#354262
BeyoMag™ Anti-Flag Magnetic Beads			Beyotime	CAT#P2115
Lipo8000™ Transfection Reagent			Beyotime	CAT#C0533
Protein A+G Agarose			Beyotime	CAT#P2012
Modified Hematoxylin-Eosin (HE) Stain Kit			Solarbio	CAT#G1121
DAB Kit			MXB biotechnologies	CAT#DAB-0031
ElivisionTM plus Polyer HRP (Mouse/Rabbit) IHC Kit			MXB biotechnologies	CAT#Kit-9902
Ready-to-use normal goat serum			BOSTER	CAT#AR0009
Glycogen Periodic Acid Schiff (PAS/Hematoxylin) Stain Kit			Solarbio	CAT#G1281
MolPure Cell RNA Kit			Yeasen	CAT#19231ES50
Hifair® Ⅲ 1st Strand cDNA Synthesis SuperMix for qPCR (gDNA digester plus)			Yeasen	CAT#11141ES60
Hieff UNICON® Universal Blue qPCR SYBR Green Master Mix			Yeasen	CAT#11184ES25
Mounting Medium, antifading (with DAPI)			Solarbio	CAT#S2110
MycoAlert mycoplasma detection kit			Lonza	CAT#LT07-218
Pierce(tm) BCA Protein Assay Kit			Thermo Fisher	CAT#23227
**Antibodies**		**Company**	**CAT#**	**RRID**
Rabbit polyclonal anti-beta Actin		Affinity	CAT#AF7018	RRID: AB_2839420
Mouse monoclonal anti-VDR (D-6)		Santa Cruz	CAT#sc-13133	RRID: AB_628040
Mouse monoclonal anti-YY1(H-10)		Santa cruz	CAT#sc-7341	RRID: AB_2257497
Mouse monoclonal anti-Vitamin D binding protein (DBP)		Proteintech	CAT#66175-1-Ig	RRID: AB_2881570
Mouse monoclonal anti-E-cadherin		Proteintech	CAT#60335-1-Ig	RRID: AB_2881444
Rabbit polyclonal anti-VE-Cadherin		Affinity	CAT#AF6265	RRID: AB_2835123
Mouse monoclonal anti-Vimentin		Proteintech	CAT#60330-1-Ig	RRID: AB_2881439
Rabbit polyclonal anti-Twist1		Proteintech	CAT#25465-1-AP	RRID: AB_2880093
Rabbit polyclonal anti-MMP2		Affinity	CAT#AF0577	RRID: AB_2834154
Rabbit polyclonal anti-MMP9		Affinity	CAT#AF0220	RRID: AB_2833350
Rabbit polyclonal anti-CD31		ZENBIO	CAT#347526	N/A
*In vivo* Mab anti-mouse PD-1(CD279)		Bio X Cel	CAT#BE0273	RRID: AB_2687796
Rabbit polyclonal anti-Fibronectin 1		Proteintech	CAT#15613-1-AP	RRID: AB_2105691
Mouse monoclonal anti-SERPINE2		Proteintech	CAT#66203-1-Ig	RRID: AB_2881594
Rabbit IgG		Beyotime	CAT#A7016	RRID: AB_2905533
Mouse IgG		Beyotime	CAT#A7028	RRID: AB_2909433
Goat Anti-Rabbit IgG (H+L) HRP		Affinity	CAT#S0001	RRID: AB_2839429
Goat Anti-Mouse IgG (H+L) HRP		Affinity	CAT#S0002	RRID: AB_2839430
CoraLite 488-conjugated Goat Anti-Mouse IgG(H+L)		Proteintech	CAT#SA00013-1	RRID: AB_2810983
YF 633-Phalloidin		US Everbright	CAT#YP0053S	N/A
**Plasmids**		**Company**		**Vector backbone**
GC_OHu14110D_pcDNA3.1+/C-(K)-DYK		Genscript		pcDNA3.1(+)
ko GC_eSpCas9-2A- Puro (PX459) V2.0		Genscript		pX459
PGL3-Basic-GC-promoter		TsingkeBiotechnology		PGL3-Basic
PGL3-Basic-VE-cadherin-promoter		TsingkeBiotechnology		PGL3-Basic
pRL-TK		TsingkeBiotechnology		PRL-TK
GC-ΔTbd_pcDNA3.1+/C-(K)-DYK		TsingkeBiotechnology		pcDNA3.1(+)
VDR_pcDNA3.1+/C-(K)-DYK		Genscript		pcDNA3.1(+)
Ko VDR_eSpCas9-2A-Puro (PX459) V2.0		Genscript		pX459
Twist1_pcDNA3.1+/C-(K)-DYK		TsingkeBiotechnology		pcDNA3.1(+)
YY1_pcDNA3.1+/C-(K)-DYK		TsingkeBiotechnology		pcDNA3.1(+)
ko YY1_eSpCas9-2A- Puro (PX459) V2.0		Genscript		pX459
**CRISPR/Cas9-based knock out**				
**sgRNA**		**Targeting sgRNA Sequence (5'-3')**		**Vector backbone**
Ko VDR_eSpCas9-2A-Puro (PX459) V2.0		ACTTTGACCGGAACGTGCCC		PX459
Ko GC_eSpCas9-2A- Puro (PX459) V2.0		ACCCTGACTGCTATGACACC		PX459
ko YY1_eSpCas9-2A- Puro (PX459) V2.0		GATGTAGAGGGTGTCGCCCG		PX459
**Oligonucleotides**		**Company**		
Primers for GAPDH Forward: ACAACTTTGGTATCGTGGAAGG Reverse: GCCATCACGCCACAGTTTC		TsingkeBiotechnology		
Primers for CDH1Forward: ATTTTTCCCTCGACACCCGATReverse: TCCCAGGCGTAGACCAAGA		TsingkeBiotechnology		
Primers for CDH5Forward: AAGCGTGAGTCGCAAGAATG Reverse: TCTCCAGGTTTTCGCCAGTG		TsingkeBiotechnology		
Primers for MMP2Forward: TACAGGATCATTGGCTACACACCReverse: GGTCACATCGCTCCAGACT		TsingkeBiotechnology		
Primers for MMP9Forward: TGTACCGCTATGGTTACACTCGReverse: GGCAGGGACAGTTGCTTCT		TsingkeBiotechnology		
Primers for GC promoterForward: CCCAGTGGCACGTTTGAAC Reverse: CTGGTGTCATAGCAGTCAGGG		TsingkeBiotechnology		
**Biological samples**		**Source**		
HCC samples for patient-derived xenografts		Tianjin Medical University Cancer Institute and Hospital		
**Experimental models: Cell lines**		**Source**		**CAT#**
SK-HEP-1		Keygen Biotech		CAT#KG064
Huh-7		Keygen Biotech		CAT#KG435
SNU-387		CELLCOOK		CAT#CC0112
PLC-PRF-5		Keygen Biotech		CAT#KG068
MHCC-97H		ZQXZbio		CAT#ZQ0020
MHCC-97H-LUC		ZQXZbio		CAT#LZQ0018
SNU-387-LUC		UBIGENE		CAT#YC-B001-Luc-P
**Experimental models: Organisms/strains**		**Source**		**RRID**
BALB/c-nude mice		Beijing Vital River Laboratory Animal Technology		
NOD/SCID mice		Beijing Vital River Laboratory Animal Technology		
C57BL/6 GC flox/+ mice		Shanghai Model Organisms Center		RRID: IMSR_NMCKO-2117028
C57BL/6 Alb-Cre mice		Shanghai Model Organisms Center		
**Software and algorithms**	**Source**	**RRID**		
**ZEN**	**Zeiss**			
IGV	IGV			
R 4.1.3	R Foundation	RRID:SCR_001905		
ImageJ	Open-source processing software	RRID:SCR_003070		
GraphPad Prism 9.0.0	GraphPad	RRID:SCR_002798		
Pymol	Schrödinger, LLC	RRID:SCR_000305		
ClusPro 2.0	SciCrunchRegistry	RRID:SCR_018248		
Living Imaging 4.5.5	Perkinelmer	RRID:SCR_014247		
**Other**				
FigDraw for Graphical abstract		ID: TUWOT188d8		
Zeiss LSM800 with Airyscan	Zeiss			
