# Vitamin D binding protein (VDBP) hijacks twist1 to inhibit vasculogenic mimicry in hepatocellular carcinoma: Erratum

**DOI:** 10.7150/thno.110724

**Published:** 2025-02-15

**Authors:** Lu-ning Qin, Heng Zhang, Qing-qing Li, Ting Wu, Shan-bin Cheng, Kai-wen Wang, Yue Shi, Hao-ran Ren, Xue-wu Xing, Cheng Yang, Tao Sun

**Affiliations:** 1 State Key Laboratory of Medicinal Chemical Biology and College of Pharmacy, Nankai University, Tianjin, China.; 2 Tianjin International Joint Academy of Biomedicine, Tianjin, China.; 3 Department of Orthopedics, Tianjin First Central Hospital, Tianjin, China.

In the originally published version of this article, when using graphic software for image composition and labeling the groups when creating the statistical chart, we linked the pictures incorrectly in Fig 2, and mistakenly labeled Fig 6K and Fig 7B. We have corrected these errors. This issue does not affect the conclusion of this article. The authors regret this error and sincerely apologize to the Journal and its readers.

## Figures and Tables

**Figure A:**
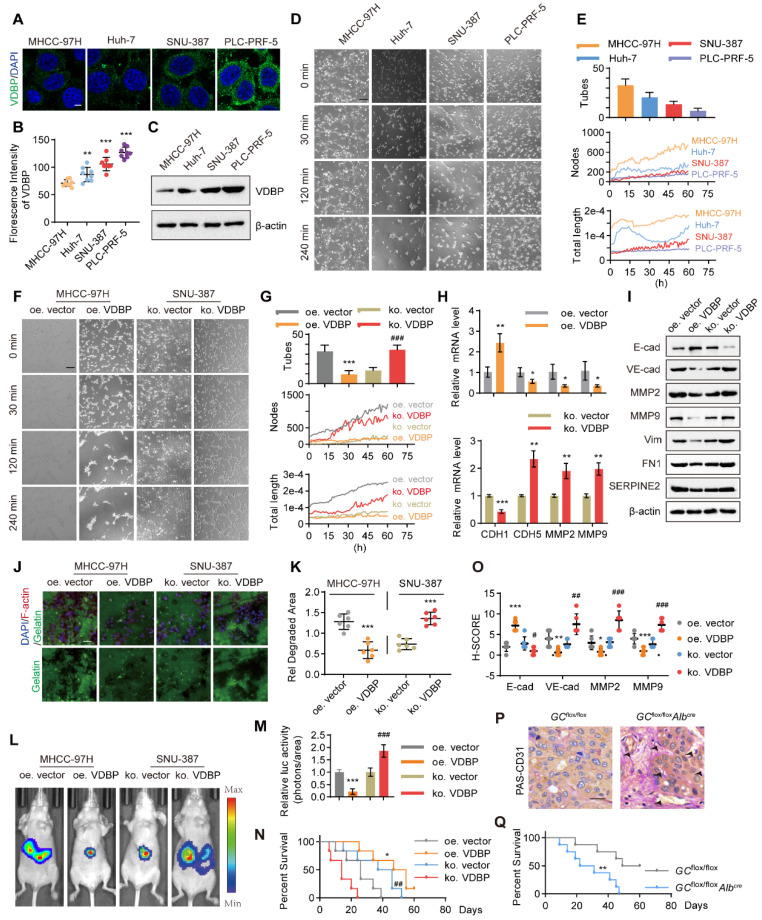
Corrected figure for original Figure 2.

**Figure B:**
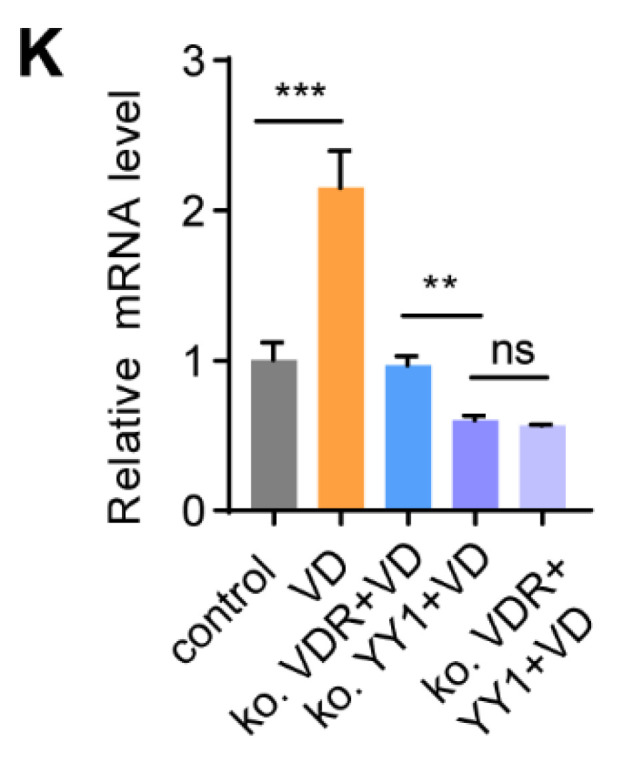
Corrected figure for original Figure 6K.

**Figure C:**
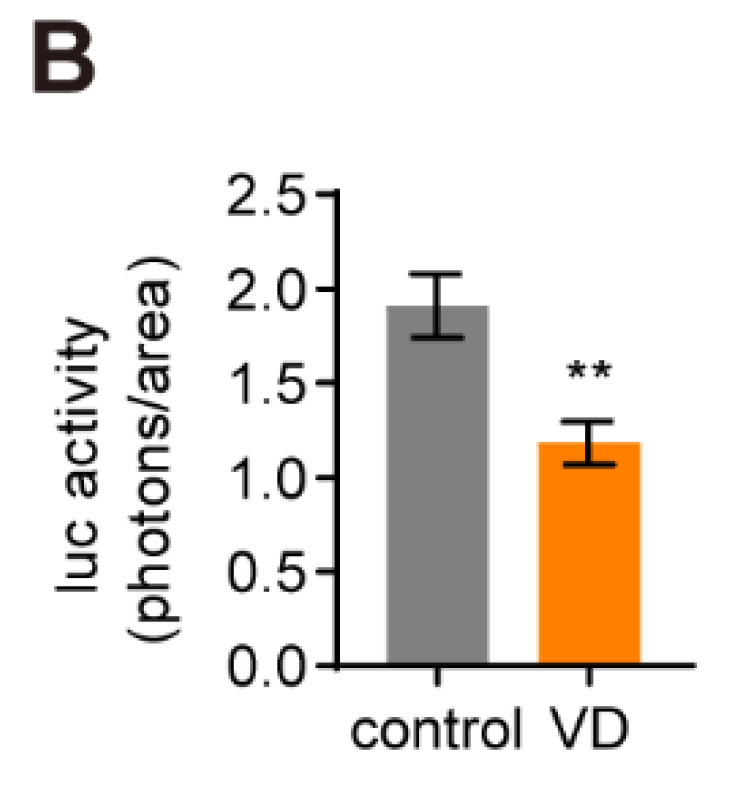
Corrected figure for original Figure 7B.

