# Supplementary material for: Vitamin D binding protein (VDBP) hijacks twist1 to inhibit vasculogenic mimicry in hepatocellular carcinoma
Source: Theranostics. 2024 Jan 1;14(1):436–50. doi: 10.7150/thno.90322 (PMC10750215; doi:10.7150/thno.90322)
Supplement: Supplementary file 1 — Supplementary figures and methods. [file thnov14p0436s1.pdf]

Supplementary materials

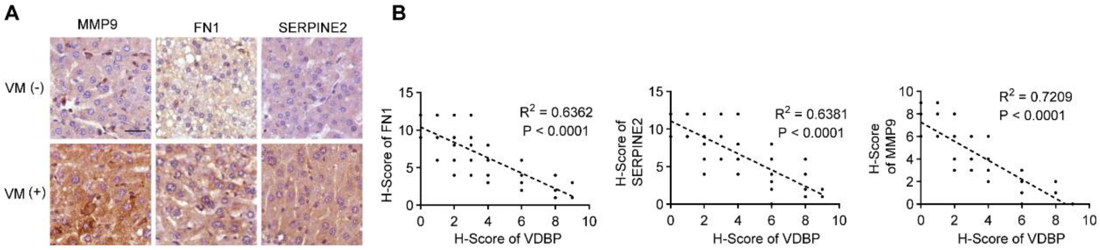

Figure S1. Raw images of IHC staining of FN1 and SERPINE2.

(A) Representative images of IHC staining of MMP9, FN1 and SERPINE2 proteins in VM-negative and VM-positive HCC samples. Scale bar,40 μm. **(K)** Correlation analysis of VDBP with MMP9, FN1 and SERPINE2 respectively. n = 75. Statistics were calculated on biological replicates with simple linear regression.

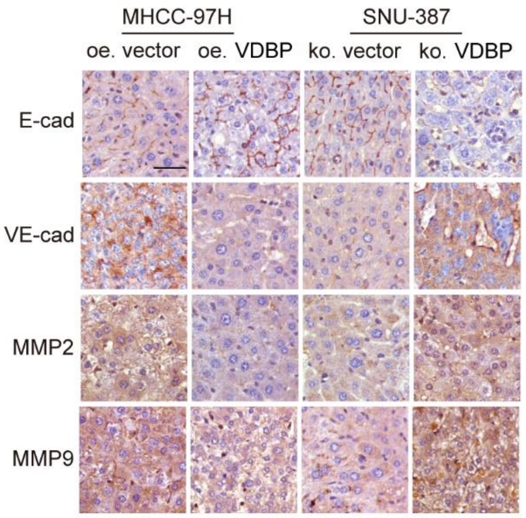

Figure S2. Raw images of IHC staining of E-cad, VE-cad, MMP2 and MMP9, related to Fig 2O.

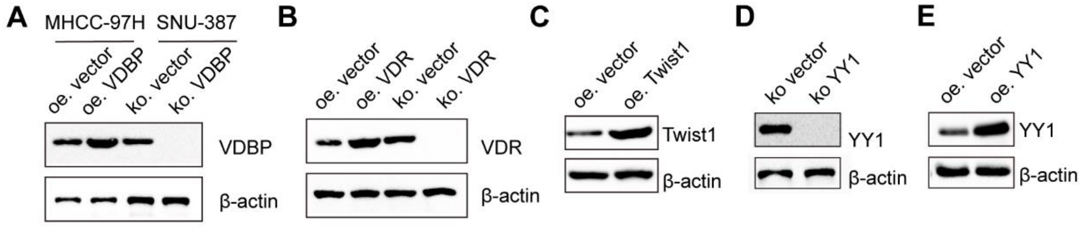

Figure S3. Western blotting for verifying knockout of VDBP, VDR and YY1 and overexpression of VDBP, VDR and Twist1.

(A) Confirmation of VDBP oe and VDBP ko in the MHCC-97H-LUC cell line and SNU-387-LUC cell line MHCC-97H-LUC respectively by Western blot analysis. **(B-E)** Confirmation of VDR oe,VDR ko,Twist1 oe， YY1 ko and YY1 oe in the MHCC-97H cell line by Western blot analysis. β-actin protein was used as a loading control. Unless otherwise specified, cells with overexpressed proteins were collected for verification 48 h after transfection, and cells with knockout proteins were selected for stable transfection with complete medium containing puromycin after transfection.

Methods

Cell culture

The human HCC cell lines SK-HEP-1, Huh-7, and PLC-PRF-5 were obtained from Nanjing Keygen Biotech. SNU-387 was obtained from the Guangzhou CELLCOOK, the SNU-387-LUC was purchased from UBIGENE and the cell lines MHCC-97H and MHCC-97H-LUC were purchased from Shanghai Zhong Qiao Xin Zhou Biotechnology. All cell lines were subjected to STR analysis, and the cell line mycoplasma-free status was verified using the MycoAlert mycoplasma detection kit (Lonza, Cat#LT07-218). SK-HEP-1, SNU-387 and SNU-387-LUC cells were cultured in RPMI-1640 medium (Keygen Biotech), and Huh-7, PLC-PRF-5, MHCC-97H and MHCC-97H-LUC cells were cultured in Dulbecco’s modified Eagle’s medium (Keygen Biotech) supplemented with 10% (v/v) fetal bovine serum (Thermo Fisher Scientific, USA) and 1% penicillin-streptomycin solution (Keygen Biotech) in a humidified environment containing 5% CO_2 at 37 °C. Complete cell identification was provided by each manufacturer.

IHC and HE staining

The paraffin-embedded tumor tissue was cut into 4 μm thick slices, and baked in an oven at 60 °C for 4-6 h. After dewaxing and hydration at room temperature, a sodium citrate antigen retrieval solution was used for antigen retrieval. A 3% hydrogen peroxide and goat serum blocking solution was then added to inactivate endogenous peroxidase and block non-specific sites, respectively. Samples were incubated overnight at 4 °C with primary antibodies. After washing with PBS, the samples were incubated with an ElivisionTM plus Polyer HRP (Mouse/Rabbit) IHC Kit (MXB, Cat#Kit-9902) for 30 min at room temperature. A DAB Chromogenic Kit (MXB, Cat#DAB-0031) was used for color development, and hematoxylin was used for counterstaining. The tissues were dehydrated, sealed with neutral gum, and photographed under a microscope after drying. The images were obtained under the microscope, and were performed for IHC scoring (H-score). Specifically, the staining degree (0-3 points) and the positive rate (0-4 points) of the IHC sections were scored separately and multiplied together to obtain a

comprehensive score (0-12 points). Staining intensity was scored as follows: 0 for no staining, 1 for light yellow, 2 for brownish yellow, 3 for brownish brown; 0 points for no positive tumor cells, 1 point for 0.01%-25%, 2 points for 25.01%-50%, 50.01%-75% is 3 points, and 75% or greater positive tumor cells is 4 points [1, 2]. The IHC antibodies used were VE-Cadherin Antibody (Affinity, Cat#AF6265), E-Cadherin Antibody (Proteintech, Cat#60335-1-Ig), Vimentin Antibody (Proteintech, Cat#60330-1-Ig), MMP2 Antibody (Affinity, Cat#AF0577) and MMP9 Antibody (Affinity, Cat#AF0220). Sections were stained with an HE staining kit (Solarbio, Cat#G1121), deparaffinized and permeabilized with xylene, dehydrated with graded ethanol, washed with tap water, and then stained with hematoxylin and eosin at room temperature. After staining, the sections were dehydrated, sealed, and observed under a microscope.

PAS-CD31 staining

PAS staining and CD31 IHC were used to assess the presence and degree of mimicry in tumor tissues. Briefly, after IHC staining with a Rabbit anti-CD31 monoclonal antibody (ZENBIO, Cat#347526), tissue sections were treated with sodium periodate for 10 min, washed with distilled water for 5 min, and then incubated with PAS (Solarbio, Cat#G1281) for 15 min. All sections were counterstained with hematoxylin, dehydrated, and mounted.VM is characterized by a luminal structure lined by tumor cells, negatively stained for CD31, and positively stained for PAS; whereas the endothelial microvascular marker is positively stained for CD31, and positively stained for PAS.

Immunofluorescence staining

The cell medium was discarded, cells were washed with pre-cooled PBS, fixed with 4% paraformaldehyde at room temperature for 20 min, and wells were blocked with an immunostaining blocking solution (Beyotime, Cat#P0260) for 30 min at room temperature. After washing with PBS, cells were incubated with a primary antibody (VDBP Antibody, Proteintech, Cat#66175-1-Ig) at room temperature for 2 h, then with the corresponding fluorescent secondary antibody (Alexa Fluor 488, Proteintech, Cat#SA00013-1) at room temperature, and incubated for 2 h. The slides were mounted using DAPI-containing anti-fluorescence fading mounting medium (Solarbio,

Cat#S2110). Finally, laser confocal microscopy (LSM 800 with airscan; Zeiss) was used for observation and analysis.

Western blot analysis

Cell lysates were collected at 4 °C and protein levels were quantified using a standard BCA assay kit (Thermo Fisher, Cat#23227). Proteins were resolved on 10% SDS-PAGE gels and transferred to PVDF membranes (Millipore). After blocking in 5% skim milk for 2 h, anti-E-cadherin (Proteintech), VE-Cadherin (Affinity), MMP2 (Affinity), MMP9 (Affinity), Vimentin (Proteintech), FIN1 (Proteintech, Cat# 15613-1-AP), SerpinE2 (Proteintech, Cat#66203-1-Ig), β-actin (Affinity, Cat#AF7018), Twist1 (Proteintech, Cat#25465-1-AP), VDBP (Proteintech) were added before incubation overnight at 4 °C. The membrane was then washed 3 times with TBST, 10 min each time, then incubated with a horseradish peroxidase-conjugated goat anti-rabbit IgG (Affinity, Cat# S0001) or a goat anti-mouse IgG (Affinity, Cat# S0002) secondary antibody for 2 h at room temperature. Finally, an enhanced ECL luminescent fluid was used to detect protein expression with chemiluminescent imaging.

Total RNA isolation and qRT-PCR

The RNA extraction kit (Yeasen, Cat#19231ES50) was used according to the reagent manufacturer's instructions to extract the total RNA of each group of cells after treatment and measure the concentration of RNA. The Strand cDNA Synthesis SuperMix master mix (Yeasen, Cat#11141ES60) was used to reverse transcribe the RNA into cDNA. Fluorescent quantitative PCR was performed using qPCR SYBR Green Master Mix Kit (Yeasen, Cat#11184ES25). The $2^{-\Delta\Delta CT}$ method was used to quantify relative gene expression compared to GAPDH as an internal control. qRT-PCR primers are listed in the table.

Dual-Luciferase reporter gene assay

For the luciferase activity assay, the PGL3-promoter luciferase reporter plasmid, the pRL-TK luciferase reporter vector plasmid, and the corresponding transcription factor plasmid were co-transfected into cells for 24 h using the Lipo8000 transfection reagent. After the cells were collected, the luciferase activity was analyzed using the Dual Luciferase Reporter Gene Assay Kit (Beyotime, Cat#RG088s) according to the

manufacturer's guidelines, and the firefly luciferase and renilla luciferase were detected using a multi-function microplate reader (Thermo). Each experiment was repeated 3 times.

In vitro tube formation assay

To assess the VM ability of HCC cells, a matrigel-based *in vitro* tube formation assay was applied. Briefly, Matrigel (CORNING, Cat#354262) and serum-free medium were mixed at a ratio of 1:2 and transferred to a 24-well plate. The bottom of the well was covered. The plates were then placed at 37 °C for 2 h until the Matrigel solidified. Cells were resuspended in a serum-containing medium, seeded on the surface of the Matrigel at a density of 3×10^5 cells/mL, incubated in a 37 °C incubator for 1 h, and subjected to live cell dynamic imaging under a fluorescent microscope Qi2 (Nikon). The images were digitized with ImageJ and the AngiogenesisAnalyzer.ijm and AutoMeasure.ijm scripts [3] were employed to analyze the time series images of the VM.

Gelatin degradation assay

Porcine skin gelatin (Thermo Fisher, Cat#G13187) was diluted with 2% sucrose in PBS to a final gelatin concentration of 0.2 mg/mL. The working solution was protected from light, and heated to 60 °C to evenly cover the slides. After drying, 1 mL of pre-cooled glutaraldehyde solution was added and the solution was incubated on ice for 15 min. Coverslips were washed three times with PBS at room temperature. 1 mL of freshly prepared sodium borohydride solution was then added, and the solution was incubated at room temperature for 3 min. After inoculating the cells and culturing them for 24 h, the cells were fixed and stained with F-Actin using YF 633-Phalloidin (US Everbright, Cat#YP0053S). Finally, the slides were mounted with DAPI and observed and analyzed using a laser confocal microscope (LSM 800 with airyscan).

Pull down and silver staining

In MHCC-97H cells, we studied the regulatory effect of VDBP on VM in HCC and explored the interacting proteins of VDR. MHCC-97H cells were subjected to an overexpressed vector, GC_pcDNA3.1+/C-(K)-DYK or VDR_pcDNA3.1+/C-(K)-DYK for 48 h, and then a 0.3% NP containing protease inhibitors -40 lysis buffer (Sigma, USA) to obtain lysates of cells after different treatments. The cell extract was

incubated with anti-Flag tag affinity magnetic beads (Beyotime, Cat#P2115) for 12 h at 4 °C. After the binding was complete, the beads were washed with pre-cooled 0.1% NP-40 lysis buffer, and the elution liquid was collected. Electrophoresis was performed with 10% SDS-PAGE, followed by staining with a rapid silver staining kit (Beyotime, Cat#P0017S) which was terminated after the ideal expected protein band appeared, and the SDS-PAGE gel was photographed. Selected bands were excised from the gel, destained, and dehydrated, followed by trypsin digestion, and finally, the extracted peptides were vacuum-dried and concentrated for protein identification by mass spectrometry (MS).

Co-IP assay

For immunoprecipitation, 50 μL of Protein A+G Agarose (Beyotime, Cat# P2012) was incubated with specific antibodies: Twist1 (Proteintech), VDBP (Proteintech), VDR (Santa Cruz, Cat#sc-13133) or YY1 (Santa Cruz, Cat#sc-7341) at 4 °C consecutively and then incubated overnight with rotation. IP lysate was used to lyse and collect protein samples. An appropriate amount of protein samples was taken and incubated with antibody-immobilized beads overnight at 4 °C. After incubation, the beads were washed three times with a cold lysis buffer. Precipitated proteins were resuspended in 2×SDS-PAGE buffer and boiled at 99 °C for 10 min to separate the precipitated proteins from the beads, and the boiled proteins were analyzed by Western blotting.

Duolink in situ proximity ligation assay

Duolink *in situ* PLA was performed using the Duolink In Situ Priming Kit Mouse/Rabbit (Sigma Aldrich, Cat#DUO92001, Cat#DUO92005, Cat#DUO92007) according to the manufacturer's protocol. Briefly, cells were plated on glass slides, washed three times with PBS, and fixed in 4% formaldehyde in PBS for 10 min. Cells were permeabilized in immunostaining permeabilization buffer containing Triton X-100 for 5 min and blocked with 3% BSA in PBS for 60 min at 37 °C. After blocking, cells were incubated with primary antibodies in PBS containing 1% BSA overnight at 4 °C, followed by incubation with corresponding PLA probe-conjugated secondary antibodies at 37 °C for 60 min in the dark. Cells were washed three times in a wash solution. Finally, the cells were stained with DAPI, and the Duolink signal was detected

using a laser confocal microscope (LSM 800 with airyscan). For the PLA of the *in vitro* tube formation assay, the cells are first plated on matrigel to form a tube, and then the subsequent PLA experiment steps are performed as described above. For PLA of tissue slices, the tissue slices were permeabilized, dehydrated, and antigen retrieved, and then PLA experiments were performed according to the manufacturer's protocol.

CHIP and CHIP-reCHIP qPCR analysis

ChIP assay was performed using the Chromatin Immunoprecipitation Kit (Beyotime, Cat#P2078) following the manufacturer's guidelines. Briefly, the cells of different groups were cross-linked with 1% formaldehyde solution for 10 min at room temperature, washed with pre-cooled PBS, and then lysed in SDS buffer containing a protease inhibitor cocktail. The lysate was collected by centrifugation at 4 °C. Each primary antibody Twist1(Proteintech), YY1(Santa cruz), VDR (Santa cruz), or mouse IgG (Beyotime, Cat#A7028) was added for immunoprecipitation of cross-linked protein/DNA. Immunoprecipitated and eluted DNA was column purified and subsequently amplified and detected by qPCR. For ChIP-reChIP, the first ChIP was performed with the YY1 antibody until the wash step. The immunoprecipitated protein-DNA complexes in ChIP-reChIP elution buffer (2 mM EDTA, 500 mM NaCl, 0.1% SDS, 1% NP40, supplemented with protease inhibitor cocktail) were incubated for 30 min at 37 °C. The isolated supernatant was diluted 20-fold and subjected to a second ChIP using VDR antibody or IgG. DNA obtained from two consecutive ChIPs was used as a PCR template for amplification.

CUT&Tag assay and sequencing

CUT & Tag assay was performed according to the manufacturer's instructions (Yeasen, Cat#12598ES12). In short, SNU-387 cells were treated with VD 500 nM or DMSO for 48 h, and 100,000 cells were collected and lysed in each group. The cell lysate with the activated concanavalin A-coated magnetic beads was incubated at room temperature for 20min, incubated with VDR antibody or YYI antibody for 2 h, incubated with mouse IgG for 1 h, and then incubated with pA /G-Tn5 transposase for 1 h, and lastly, the transposase was activated and cells were split at 37 °C for 1 h. After incubation with proteinase K for 30 min at 55 °C, DNA was extracted for CUT&Tag assay. DNA

libraries were prepared by PCR according to the manufacturer's protocol (Yeasen).

Animal studies

According to the Declaration of Helsinki, animal experiments and tumor specimen studies were performed after approval of the ethics committee of Nankai University (approval no.2022-SYDWLL-000095). All experimental protocols were approved by the Animal Care and Use Committee of Tianjin International Biomedical Joint Research Institute. All animals were maintained in a specific pathogen-free animal care facility according to institutional guidelines, and animals were allowed to acclimate for at least 5 days before initiation of the study. All animals were euthanized after the experiment.

In vivo orthotopic implantation model

Female BALB/c nude mice aged 4-6 weeks were used to establish an orthotopic liver tumor model and to observe tumor metastasis. Briefly, MHCC-97H-LUC cells or SNU-387-LUC cells transfected with GC-pcDNA3.1+/C-(K)-DYK, vector, ko vector or ko GC_eSpCas9-2A- Puro (PX459) V2.0 SNU-387 cells, oeTwist1, oeTwist1+GC-pcDNA3.1+/C-(K)-DYK, oeTwist1+GC-pcDNA3.1 or +/C-(K)-DYK-ΔTbd. The suspension was orthotopically injected into the left liver lobe of nude mice in 100 μL of PBS. For cells with overexpressed genes, transient transfection for 48 h was sufficient. To select stably transfected knockout cells, cells were maintained in a complete medium supplemented with puromycin (1.5 μg/mL) to generate permanent knockout cells. The IVIS imaging system (Perkin Elmer) was used for bioluminescence imaging studies in mice. D-Luciferin potassium (150 ug/g, meilunstar, Cat#115144-35-9) was injected intraperitoneally into the mice, and the mice were anesthetized when the peak fluorescein uptake was reached 10 min after the injection and then imaged, and the bioluminescent intensity was calculated using Living Image software. For the VD treatment experiment, the MHCC-97H-LUC transplanted mice were randomly divided into two groups, the control group and the VD group, respectively. The daily drinking water of the two groups of mice was supplemented with absolute ethanol or VD3 1500 IU/kg (Med Chem Express, Cat#HY-15398) dissolved in absolute ethanol. The treatment was administered continuously for 2 months, and at the same time, the tumor growth in mice was detected using the *in vivo* imaging system. Finally, the livers

of mice in each group were collected for subsequent IHC experiments.

Hepatocyte-specific GC deletion mouse model

C57BL/6 *GC* $^{flox/flox}$ mice and *Alb* ^{Cre} mice were purchased from the Shanghai Model Organisms Center, and hepatocyte-specific *GC* deletion mice (*GC* $^{flox/flox}$ *Alb*^{Cre}) were created by crossing *GC* $^{flox/flox}$ mice and Alb ^{Cre} mice. Littermates *GC* $^{flox/flox}$ Cre- mice were used as controls. Mice were finally euthanized, and liver tissues were fixed in formalin for subsequent PAS-CD31 double-staining experiments.

PDX mouse model

Eight fresh surgical tumor tissues (F0, hepatocellular carcinoma) without any other treatment were collected from 8 patients immediately after the operation in Tianjin Medical University Cancer Institute and Hospital and Tianjin First Central Hospital, ect. Written informed consent was obtained from each patient. Under sterile conditions, the tumor tissue was cut into pieces and implanted subcutaneously in the right axillary of NOD/SCID mice. These mice at the transplantation stage were called F1 mice. By palpating the skin at the tumor site, we selected mice with tumor nodules and started measuring tumor volumes. When the tumor size reached 100-200 mm^3, sample F1 was divided into small pieces for passage *in vivo* to construct F2 and F3 tumors. When the tumor volume of the F3 PDX model was 100-200 mm^3, mice bearing different tumor types were randomly divided into a control group, anti-PD-1 group, and anti-PD-1+VD group, with 6 mice in each group. Absolute ethanol was added to the daily drinking water of mice in the control group. For the anti-PD-1 group, 200 μg of α-PD-1 monoclonal antibody (Bio X Cel, Cat#BE0273) was injected intraperitoneally to treat tumor-bearing mice. For anti-PD-1+VD group, mice were injected intraperitoneally with 200 μg of α-PD-1 monoclonal antibody, and VD3 1500 IU/kg dissolved in absolute ethanol was added to the daily drinking water of the mice. After two months of treatment, the livers of mice in each group were collected for HE and IHC experiments.

References

1. Allred DC, Harvey JM, Berardo M, Clark GM. Prognostic and predictive factors in breast cancer by immunohistochemical analysis. Modern pathology : an official journal of the United States and Canadian Academy of Pathology, Inc. 1998; 11: 155-68.
2. Chen Z, Wang HW, Wang S, Fan L, Feng S, Cai X, et al. USP9X deubiquitinates ALDH1A3 and maintains mesenchymal identity in glioblastoma stem cells. The Journal of clinical investigation. 2019; 129: 2043-55.
3. Li Z, Zhang H, Sun Y, Feng Z, Cui B, Han J, et al. Live-cell imaging-based dynamic vascular formation assay for antivascular drug evaluation and screening. iScience. 2023; 26: 106721.
